# Burden of atrial fibrillation and its attributable risk factors from 1990 to 2019: An analysis of the Global Burden of Disease study 2019

**DOI:** 10.3389/fcvm.2022.997698

**Published:** 2022-10-26

**Authors:** Shangbo Xu, Yangbo Chen, Rui Lin, Weipeng Huang, Haoyue Zhou, Yongjian Lin, Mingwei Xu

**Affiliations:** Department of Cardiology, Jieyang People’s Hospital, Jieyang, China

**Keywords:** Global Burden of Disease, atrial fibrillation, epidemiology, trend, risk factor

## Abstract

**Background:**

Understanding the pattern and trend of the atrial fibrillation (AF) burden are essential for developing effective preventive strategies. The purpose of this study was to estimate AF burdens and risk factors in 204 countries and territories between 1990 and 2019.

**Materials and methods:**

Data were extracted from the Global Burden of Disease 2019, including incidence, death, disability-adjusted life-years (DALYs), and the attributable risk factors. In order to quantify changes in the age-standardized incidence rate (ASIR), age-standardized death rate (ASDR), and age-standardized DALY rate between 1990 and 2019, the estimated annual percentage change (EAPC) was used. Also, AF burden was assessed in relation to the Socio-demographic Index (SDI).

**Results:**

Globally, there were 4,720,324 incident cases, 117,038 deaths and 8,393,635 DALYs in 2019. There were no significant changes in ASIR, ASDR, or age-standardized DALY rates from 1990 to 2019. Although the burden and trend of AF varied in different regions and countries, the ASIR, ASDR and age-standardized DALY rate were positively correlated with SDI. Furthermore, the burden of AF was higher in males and elderly. The age-standardized DALY rate worldwide was primarily attributable to high systolic blood pressure, followed by high body-mass index, alcohol use, smoking, diet high in sodium and lead exposure.

**Conclusion:**

AF remained a major public health challenge worldwide, with substantial variation at regional and national levels. There is an urgent need to increase public awareness about AF risk factors and to bring about cost-effective interventions for AF in order to reduce its future burden.

## Introduction

Atrial fibrillation (AF) is the most common arrhythmia clinically. AF prevalence is increasing globally at a phenomenal rate, and it is clearly associated with increased heart failure, stroke, and all-cause mortality (1, 2). Furthermore, AF is an important cause of substantial healthcare costs. AF hospitalization has increased exponentially in recent years, leading to a growing economic burden on the healthcare system (3, 4). Therefore, regular assessment of the AF current burden and attributable risk factors is important to formulate preventive and control strategies.

Previous studies have reported the burden of AF from 1990 to 2017 based on data from the Global Burden of Disease (GBD) 2017 (5, 6), but more recent assessments are urgently needed. Association of AF burden with Socio-demographic Index (SDI) of countries was not examined in previous study. The GBD 2019, with incorporating newly available datasets and methodological refinements, provides an opportunity to comprehensively estimate the pattern and trend of the AF burden. In this study, we examined the data from the GBD 2019 to present the temporal and geographical trends in the incidence, death, disability-adjusted life years (DALYs) in terms of counts and age-standardized rate (ASR) from 1990 to 2019. Moreover, estimated annual percentage change (EAPC) was used to quantify the trends of ASRs from 1990 to 2019. In addition, the association between risk factors and AF among different regions and age groups were also analyzed. Risk factors assessments play a vital role in formulating and implementing disaster risk reduction policies. As such, the aim of this study was to examine the assessments of the distribution, burden, and trends of AF at global, regional, national levels, which could provide important epidemiological information for policy makers.

## Materials and methods

### Overview

The GBD study was designed to provide comprehensive and systematic estimates of epidemic levels and trends in non-communicable and communicable diseases, and injuries worldwide. In the latest round, GBD 2019 systematically analyzed 369 diseases and injuries and 87 risk factors in 204 countries and territories, 21 regions and seven super-regions from 1990 to 2019. The general methodology used for GBD 2019 have been published elsewhere (7–9). All original data used to produce estimates for the burden of AF were publicly available on the Global Health Data Exchange^1^.

### Definitions

Paroxysmal, persistent or permanent/chronic AF and atrial flutter were all included in the GBD study (7). Diagnosis required an electrocardiogram demonstrating: (1) irregular RR intervals (without complete AV block); (2) no distinct P waves; (3) an atrial cycle length (when visible) that is usually variable and no more than 200 ms. All cardiovascular diseases coded as 427.3 in the in the 9th revision of the International Classification of Diseases and Injuries (ICD-9) and I48–I48.9 in the ICD-10 were recognized as AF in the present study.

Socio-demographic index is an overview metric that highlights the social and economic conditions for each location-year which is strongly related to health outcomes. SDI ranges between 0 and 1 (worst to best), and is determined by the geometric mean of lag distributed income per capita, total fertility rate for people under 25 years, and mean education for individuals for people over 15 years (7–9). In accordance with SDI, 204 countries and territories were divided into five quintiles: low, low-middle, middle, high-middle, and high.

Disability-adjusted life-years is the most widely used evaluation and measurement indicators of disease burden. It was calculated as the sum of the years of life lost (YLLs) and years lived with disability (YLDs). In order to estimate the YLLs, the number of deaths in each age group was multiplied by the remaining life expectancy in the age group using the GBD standard life table. To calculate the YLDs, AF was firstly divided into two severity levels: asymptomatic and symptomatic. The disability weight survey assessments were lay descriptions of the sequelae, highlighting major functional consequences and symptoms (10). There are no lay descriptions and disability weights for asymptomatic AF. A lay description of symptomatic AF was as follows “has periods of rapid and irregular heartbeats and occasional fainting.” And its corresponding disability weight was 0.224, meaning that the person would experience a 22.4% health loss during an attack compared to those in full health. YLDs for AF were calculated by multiplying the prevalence at each severity level by the disability weight at each severity level.

Three criteria were used in selecting risk factors for AF: sufficient evidence of causation, availability of exposure data, and potentially modifiable. A total of six potentially modifiable risk factors were identified, including high body-mass index (BMI), high systolic blood pressure (SBP), alcohol use, smoking, diet high in sodium and lead exposure. The definitions and methodology for quantifying the percentage contributions of the risk factors to the DALYs of AF have been published previously (8).

### Statistical analysis

The burden of AF was quantified by using the absolute number and ASR with 95% uncertainty intervals (UIs). ASR (per 100,000 persons) was used to assess the differences in the burden of AF by historical periods, genders, and locations, to avoid differences caused by the different age structures of the population over time. ASR was calculated by using following formula (11)

ASR= $\frac{\sum_{i=1}^{A}{\text{a}}_{i}\,w_{i}}{\sum_{i=1}^{A}w_{i}}$ × 100,00, [α*_*i*_*, where *i* denotes the *i*th age class, and the number of persons (or weight) (w*_*i*_*) in the same age subgroup *i* of the chosen reference standard population.] In this study, AF burden was measured by incidence, death, and DALYs. The 95% UIs were estimated based on the 2.5th and 97.5th values of random 1,000 draw-level estimates.

Moreover, the EAPC was used to illustrate the temporal trends for ASR over a specified interval. A regression line model was applied to describe the annual percentage changes in ASR, fitting to the natural logarithm of the rates, i.e., y = α + βx + ε, where y = ln (ASR), x = calendar year, and ε = error term. The EAPC was calculated as 100 × (e^β^ - 1), with 95% confidence intervals (CIs) obtained from the linear model (12, 13). R version 4.0.3 was used for all statistical analyses. A two-sided *p*-value of less than 0.05 was considered statistically significant.

## Results

### Global burden of atrial fibrillation

Globally, the incident cases of AF were increased dramatically in the past 30 years from 2,313,540 (95% UI 1,764,441–2,950,592) in 1990 to 4,720,324 (95% UI 3,644,331–5,961,597) in 2019, whereas the age-standardized incident rate (ASIR) was not significantly different between 1990 and 2019 [EAPC = 0.05(95% CI –0.03 to 0.13)] (Table 1). The likelihood of males suffering from AF was higher than that of females (63.65 vs. 53.64 per 100,000 persons in 1990, and 60.82 vs. 53.50 per 100,000 persons in 2019) (Table 1).

**TABLE 1:** The incidence of atrial fibrillation in 1990 and 2019, and its temporal trends from 1990 to 2019.

Location	Incident cases (95% UI)		ASIR per 100,000		1990–2019 EAPC of ASIR (95% CI)
	1990	2019	1990	2019	
**Global**	2313540 (1764441 to 2950592)	4720324 (3644331 to 5961597)	58.54 (44.92 to 74.24)	57.09 (44.07 to 71.90)	0.05 (–0.03 to 0.13)
**Sex**					
Male	1175409 (891356 to 1506760)	2376460 (1837669 to 3010747)	63.65 (48.98 to 80.90)	60.82 (47.14 to 76.29)	0.01 (–0.09 to 0.09)
Female	1138130 (864136 to 1459488)	2343863 (1796329 to 2973621)	53.64 (40.81 to 68.47)	53.50 (41.09 to 67.72)	0.10 (0.03 to 0.18)
**SDI**					
High	739176 (565768 to 942141)	1235202 (976182 to 1518125)	71.45 (55.34 to 90.20)	69.22 (55.37 to 85.18)	0.26 (0.08 to 0.45)
High-middle	654549 (498535 to 835088)	1194911 (912920 to 1531244)	60.34 (46.52 to 77.10)	57.97 (44.57 to 74.20)	–0.12 (–0.15 to –0.09)
Middle	517303 (393362 to 662565)	1347018 (1024382 to 1717860)	52.39 (39.69 to 66.74)	54.27 (41.16 to 69.05)	0.14 (0.08 to 0.20)
Low-middle	310215 (234333 to 398202)	733952 (558001 to 935186)	53.96 (41.04 to 68.62)	54.72 (41.51 to 69.76)	0.08 (0.06 to 0.09)
Low	91359 (68847 to 117299)	207391 (158050 to 265306)	40.67 (30.92 to 52.06)	41.97 (31.77 to 53.69)	0.12 (0.11 to 0.12)
**Region**					
Andean Latin America	2545 (1899 to 3256)	8085 (6052 to 10360)	13.63 (10.08 to 17.53)	14.84 (11.11 to 19.17)	0.43 (0.35 to 0.52)
Australasia	23330 (17604 to 29958)	42634 (32397 to 54822)	98.68 (75.18 to 125.06)	90.39 (69.71 to 114.51)	–0.24 (–0.30 to –0.18)
Caribbean	7467 (5583 to 9702)	15388 (11495 to 19899)	29.33 (22.05 to 38.08)	29.72 (22.18 to 38.44)	0.07 (0.06 to 0.09)
Central Asia	30133 (23076 to 38634)	51004 (38434 to 65518)	63.51 (48.90 to 80.87)	64.77 (49.64 to 82.66)	0.10 (0.09 to 0.12)
Central Europe	110046 (83563 to 140981)	142513 (108565 to 183042)	72.77 (55.81 to 92.66)	70.59 (54.18 to 89.82)	0.02 (–0.06 to 0.11)
Central Latin America	25021 (19024 to 32108)	73686 (55668 to 95265)	31.81 (23.94 to 41.05)	31.77 (23.84 to 41.21)	0.01 (0.00 to 0.02)
Central Sub-Saharan Africa	7014 (5235 to 9141)	16185 (12341 to 20967)	33.03 (25.06 to 42.21)	32.22 (24.45 to 41.46)	–0.10 (–0.12 to –0.09)
East Asia	463450 (350847 to 596455)	1202906 (907838 to 1545466)	55.26 (41.95 to 70.12)	57.40 (43.67 to 72.99)	0.16 (0.04 to 0.28)
Eastern Europe	193805 (147591 to 248428)	253918 (193148 to 325654)	68.40 (52.40 to 87.04)	74.40 (57.18 to 95.28)	0.35 (0.32 to 0.38)
Eastern Sub-Saharan Africa	13406 (10169 to 17334)	30351 (23226 to 38666)	19.48 (14.83 to 25.05)	19.89 (15.18 to 25.61)	0.16 (0.10 to 0.23)
High-income Asia Pacific	55018 (42071 to 70668)	77896 (59594 to 99918)	26.92 (20.67 to 34.42)	21.12 (16.28 to 27.19)	–1.58 (–1.92 to –1.23)
High-income North America	336509 (254340 to 429464)	675439 (539471 to 822528)	95.30 (73.03 to 121.00)	108.53 (87.59 to 131.44)	1.33 (0.93 to 1.74)
North Africa and Middle East	67246 (51252 to 86435)	173921 (133004 to 221692)	41.81 (31.63 to 53.41)	41.93 (31.66 to 53.56)	–0.08 (–0.12 to –0.05)
Oceania	1626 (1219 to 2085)	3970 (2996 to 5098)	57.91 (43.98 to 73.48)	59.26 (44.94 to 75.20)	0.07 (0.05 to 0.09)
South Asia	326585 (245917 to 420275)	851084 (645139 to 1089231)	60.06 (45.66 to 76.21)	61.37 (46.53 to 78.00)	0.06 (0.06 to 0.07)
Southeast Asia	150683 (114106 to 193299)	378094 (288272 to 482012)	61.29 (46.46 to 78.20)	62.77 (47.54 to 79.60)	0.09 (0.08 to 0.10)
Southern Latin America	18903 (14228 to 24342)	34681 (26262 to 44620)	40.73 (30.74 to 52.21)	41.80 (31.80 to 53.39)	0.07 (0.00 to 0.14)
Southern Sub-Saharan Africa	9957 (7639 to 12792)	20372 (15608 to 26060)	37.43 (28.33 to 48.01)	37.09 (28.17 to 47.62)	–0.02 (–0.03 to –0.01)
Tropical Latin America	37950 (28877 to 48862)	107861 (81577 to 138989)	43.44 (32.89 to 55.60)	44.80 (33.81 to 57.84)	0.55 (0.39 to 0.71)
Western Europe	402836 (309011 to 515303)	495118 (378485 to 631497)	72.79 (55.34 to 92.55)	63.01 (48.37 to 79.79)	–0.36 (–0.39 to –0.32)
Western Sub-Saharan Africa	30010 (22807 to 38669)	65217 (49907 to 83723)	35.51 (26.88 to 45.46)	36.01 (27.33 to 46.19)	0.05 (0.01 to 0.08)

UI, uncertainty interval; ASIR, age-standardized incidence rate; EAPC, estimated annual percentage change; CI, confidence interval.

The global deaths of AF increased significantly during this period, with 117,038 (95% UI 103,695–138,452) deaths in 1990 and 315,337 (95% UI 267,964–361,014) deaths in 2019, whereas the age-standardized death rate (ASDR) increased slightly by 0.04% per year (Table 2). Males had a slightly lower ASDR than females (4.13 vs. 4.37 per 100,000 persons in 1990; 4.33 vs. 4.40 per 100,000 persons in 2019) (Table 2).

**TABLE 2:** The death of atrial fibrillation in 1990 and 2019, and its temporal trends from 1990 to 2019.

Location	Death cases (95% UI)		ASDR per 100,000		1990–2019 EAPC of ASDR (95% CI)
	1990	2019	1990	2019	
**Global**	117038 (103695 to 138452)	315337 (267964 to 361014)	4.29 (3.73 to 5.09)	4.38 (3.70 to 5.05)	0.04 (0.02 to 0.06)
**Sex**					
Male	42511 (33946 to 54885)	121548 (97352 to 148261)	4.13 (3.23 to 5.37)	4.33 (3.46 to 5.33)	0.15 (0.13 to 0.17)
Female	74527 (64121 to 89412)	193789 (160729 to 224867)	4.37 (3.70 to 5.26)	4.40 (3.65 to 5.11)	-0.01 (–0.03 to 0.02)
**SDI**					
High	47157 (40195 to 59598)	109926 (86254 to 132025)	4.62 (3.93 to 5.82)	4.61 (3.67 to 5.52)	0.03 (0.01 to 0.06)
High-middle	33987 (30155 to 43292)	83844 (70923 to 100863)	4.64 (4.04 to 5.92)	4.47 (3.78 to 5.39)	-0.25 (–0.33 to -0.18)
Middle	20778 (18352 to 23539)	69793 (59854 to 80804)	3.82 (3.32 to 4.28)	4.11 (3.50 to 4.75)	0.21 (0.19 to 0.24)
Low-middle	10543 (8364 to 12755)	38423 (32144 to 44622)	3.46 (2.66 to 4.14)	4.21 (3.50 to 4.87)	0.61 (0.55 to 0.67)
Low	4502 (3016 to 5732)	13160 (9591 to 16006)	3.73 (2.42 to 4.76)	4.30 (3.08 to 5.25)	0.50 (0.47 to 0.53)
**Region**					
Andean Latin America	686 (572 to 782)	2239 (1804 to 2717)	4.41 (3.67 to 5.06)	4.32 (3.48 to 5.24)	0.10 (0.02 to 0.19)
Australasia	1522 (1251 to 1742)	4082 (3258 to 4977)	7.38 (5.96 to 8.41)	6.86 (5.45 to 8.33)	–0.44 (–0.52 to –0.36)
Caribbean	909 (768 to 1068)	2500 (2064 to 3049)	4.46 (3.72 to 5.28)	4.73 (3.91 to 5.79)	0.26 (0.16 to 0.37)
Central Asia	1284 (1025 to 1674)	2467 (2144 to 3301)	3.62 (2.82 to 4.80)	5.75 (4.95 to 7.74)	1.49 (1.33 to 1.65)
Central Europe	5141 (4569 to 6291)	10882 (9017 to 12840)	4.51 (3.93 to 5.43)	4.83 (3.99 to 5.70)	0.15 (0.10 to 0.20)
Central Latin America	2588 (2211 to 3312)	9907 (8079 to 12667)	4.46 (3.76 to 5.68)	4.56 (3.72 to 5.83)	–0.09 (–0.17 to 0.00)
Central Sub-Saharan Africa	563 (324 to 965)	1651 (1054 to 2389)	5.00 (2.85 to 8.21)	5.63 (3.55 to 8.24)	0.37 (0.28 to 0.46)
East Asia	16847 (14178 to 19679)	54066 (45777 to 62320)	4.00 (3.31 to 4.63)	3.82 (3.19 to 4.42)	–0.26 (–0.31 to –0.21)
Eastern Europe	8496 (7312 to 11898)	15982 (13463 to 20894)	4.03 (3.44 to 5.60)	4.62 (3.88 to 6.02)	0.22 (0.07 to 0.37)
Eastern Sub-Saharan Africa	1727 (1013 to 2265)	4485 (2872 to 5572)	4.23 (2.37 to 5.58)	4.69 (2.98 to 5.87)	0.34 (0.25 to 0.44)
High-income Asia Pacific	4774 (4076 to 6226)	15307 (11653 to 20615)	3.00 (2.54 to 4.03)	2.39 (1.87 to 3.19)	-0.85 (–0.95 to -0.75)
High-income North America	15093 (12600 to 18699)	36444 (29295 to 44399)	4.04 (3.36 to 4.99)	4.96 (4.01 to 6.05)	0.71 (0.63 to 0.80)
North Africa and Middle East	3463 (2708 to 3996)	10505 (8922 to 12765)	3.48 (2.67 to 4.11)	3.66 (3.07 to 4.33)	0.19 (0.10 to 0.28)
Oceania	61 (44 to 87)	171 (129 to 228)	4.10 (2.76 to 6.01)	4.25 (3.24 to 5.50)	0.17 (0.12 to 0.23)
South Asia	8753 (6602 to 11314)	36619 (28652 to 45926)	3.33 (2.41 to 4.27)	4.10 (3.18 to 5.15)	0.58 (0.46 to 0.71)
Southeast Asia	4642 (4041 to 5317)	16028 (13357 to 19305)	3.19 (2.72 to 3.64)	3.99 (3.27 to 4.75)	0.75 (0.70 to 0.82)
Southern Latin America	1783 (1505 to 2176)	4639 (3866 to 6055)	4.92 (4.13 to 5.93)	5.32 (4.44 to 6.94)	0.29 (0.19 to 0.39)
Southern Sub-Saharan Africa	582 (491 to 654)	1440 (1258 to 1587)	3.10 (2.59 to 3.50)	3.94 (3.38 to 4.37)	0.79 (0.58 to 1.00)
Tropical Latin America	2738 (2338 to 3421)	11051 (8832 to 13043)	4.78 (3.99 to 5.95)	5.03 (4.01 to 5.94)	0.50 (0.31 to 0.70)
Western Europe	33269 (28673 to 45265)	69928 (55541 to 84441)	5.77 (4.91 to 7.83)	5.76 (4.59 to 6.99)	0.06 (0.02 to 0.10)
Western Sub-Saharan Africa	2119 (1665 to 2779)	4944 (4097 to 5799)	4.50 (3.55 to 5.93)	4.75 (3.89 to 5.59)	0.08 (0.03 to 0.12)

UI, uncertainty interval; ASMR, age-standardized death rate; EAPC, estimated annual percentage change; CI, confidence interval.

The number of DALYs increased from 3,787,838 (95% UI 267,964–361,014) in 1990 to 8,393,635 (95% UI 267,964–361,014) in 2019, but the age-standardized DALY rate was not significantly different between 1990 and 2019 [EAPC = –0.03(95% CI –0.06 to 0.00)] (Table 3). In terms of sex, the age-standardized DALY rate was higher in males than in females (117.83 vs. 103.07 per 100,000 persons in 1990; 114.15 vs. 100.96 per 100,000 persons in 2019) (Table 3).

**TABLE 3:** The DALYs of atrial fibrillation in 1990 and 2019, and its temporal trends from 1990 to 2019.

Location	DALYs (95% UI)		Age-standardized DALY rate per 100,000		1990–2019 EAPC of age-standardized DALY rate (95% CI)
	1990	2019	1990	2019	
**Global**	3787838 (2961188 to 4832673)	8393635 (6693984 to 10541461)	110.00 (87.66 to 139.16)	107.13 (86.18 to 133.73)	–0.03 (–0.06 to 0.00)
**Sex**					
Male	1763682 (1336765 to 2314841)	3956017 (3087374 to 5050017)	117.83 (91.13 to 154.07)	114.15 (90.08 to 144.12)	–0.02 (–0.06 to 0.02)
Female	2024156 (1605513 to 2568325)	4437618 (3574324 to 5539382)	103.07 (82.28 to 130.38)	100.96 (81.30 to 125.99)	–0.04 (–0.06 to –0.01)
**SDI**					
High	1361228 (1075403 to 1754752)	2517229 (2012613 to 3144013)	128.62 (101.73 to 165.21)	122.64 (97.30 to 153.57)	0.03 (–0.05 to 0.11)
High–middle	1108696 (863012 to 1438273)	2214107 (1734630 to 2823347)	116.99 (92.57 to 149.81)	110.21 (86.73 to 140.14)	–0.25 (–0.28 to –0.23)
Middle	745665 (576304 to 952661)	2113471 (1653609 to 2694931)	93.38 (74.40 to 116.56)	97.90 (78.07 to 122.72)	0.17 (0.14 to 0.20)
Low-middle	421268 (319702 to 540873)	1173891 (929505 to 1468081)	91.50 (71.71 to 114.77)	101.01 (81.22 to 123.92)	0.34 (0.32 to 0.37)
Low	149125 (112096 to 190132)	370883 (291504 to 456045)	84.28 (63.03 to 105.84)	91.91 (71.89 to 111.57)	0.30 (0.27 to 0.33)
**Region**					
Andean Latin America	11255 (9596 to 13090)	33839 (28083 to 40058)	64.49 (55.40 to 74.42)	63.61 (52.67 to 75.22)	0.11 (0.04 to 0.19)
Australasia	42220 (33196 to 53571)	89702 (71277 to 113671)	183.72 (145.83 to 231.54)	168.28 (132.54 to 214.32)	–0.36 (–0.39 to –0.34)
Caribbean	18756 (15784 to 22495)	43197 (36148 to 51752)	79.40 (66.52 to 94.70)	83.27 (69.65 to 99.83)	0.19 (0.12 to 0.26)
Central Asia	46069 (34294 to 60476)	81519 (63510 to 104678)	109.82 (82.86 to 143.91)	138.35 (109.64 to 176.31)	0.82 (0.76 to 0.88)
Central Europe	188357 (145003 to 244839)	302864 (237301 to 385693)	134.74 (105.22 to 173.65)	136.04 (106.47 to 173.32)	0.07 (0.01 to 0.13)
Central Latin America	56099 (47310 to 68851)	185647 (152660 to 228151)	80.65 (68.44 to 98.18)	82.84 (68.32 to 101.95)	–0.01 (–0.05 to 0.04)
Central Sub-Saharan Africa	16297 (10915 to 24704)	40758 (28803 to 53877)	99.09 (65.94 to 148.49)	104.25 (72.95 to 138.10)	0.14 (0.07 to 0.21)
East Asia	654562 (493067 to 852620)	1793188 (1356400 to 2304890)	98.28 (76.84 to 124.34)	96.78 (75.08 to 122.56)	–0.05 (–0.13 to 0.04)
Eastern Europe	316576 (238789 to 415981)	481406 (368863 to 621279)	120.85 (92.65 to 157.62)	136.59 (104.82 to 177.07)	0.36 (0.28 to 0.44)
Eastern Sub-Saharan Africa	40780 (28065 to 51395)	94303 (66626 to 115072)	73.06 (48.45 to 92.35)	77.09 (53.33 to 94.33)	0.18 (0.09 to 0.28)
High-income Asia Pacific	124789 (101117 to 156715)	265228 (212780 to 329103)	66.35 (54.42 to 82.70)	53.53 (43.10 to 66.58)	–1.06 (–1.22 to –0.90)
High-income North America	514635 (396815 to 674657)	1059274 (837806 to 1336169)	140.63 (108.35 to 183.61)	160.18 (125.70 to 202.67)	0.89 (0.71 to 1.08)
North Africa and Middle East	106968 (84330 to 135033)	289998 (229100 to 361786)	79.91 (63.99 to 99.66)	81.61 (65.10 to 100.78)	0.01 (–0.01 to 0.04)
Oceania	2539 (1907 to 3307)	6579 (5066 to 8433)	109.88 (83.38 to 140.90)	116.58 (91.06 to 148.01)	0.23 (0.22 to 0.25)
South Asia	407485 (301692 to 533787)	1257580 (971301 to 1609102)	96.83 (73.96 to 124.55)	106.05 (82.89 to 132.86)	0.26 (0.22 to 0.31)
Southeast Asia	195204 (146920 to 256580)	544420 (417735 to 695212)	95.11 (73.30 to 122.94)	105.70 (82.22 to 133.52)	0.37 (0.34 to 0.40)
Southern Latin America	40566 (33439 to 50252)	86620 (71626 to 107603)	96.08 (79.70 to 117.41)	100.95 (83.35 to 125.77)	0.18 (0.13 to 0.22)
Southern Sub-Saharan Africa	16762 (13518 to 20930)	36881 (30132 to 45192)	71.59 (58.23 to 88.42)	79.24 (65.78 to 95.69)	0.36 (0.23 to 0.48)
Tropical Latin America	73160 (60354 to 90768)	235075 (193674 to 285763)	98.38 (81.69 to 120.63)	102.34 (84.28 to 124.34)	0.52 (0.39 to 0.65)
Western Europe	860796 (682643 to 1101763)	1347220 (1087687 to 1676380)	144.62 (114.54 to 185.07)	132.81 (105.93 to 168.11)	–0.19 (–0.22 to –0.16)
Western Sub-Saharan Africa	53962 (41706 to 68541)	118338 (94717 to 145563)	82.99 (65.49 to 103.45)	86.19 (70.00 to 103.25)	0.08 (0.05 to 0.10)

UI, uncertainty interval; DALY, disability-adjusted life-year; EAPC, estimated annual percentage change; CI, confidence interval.

**FIGURE 1:**
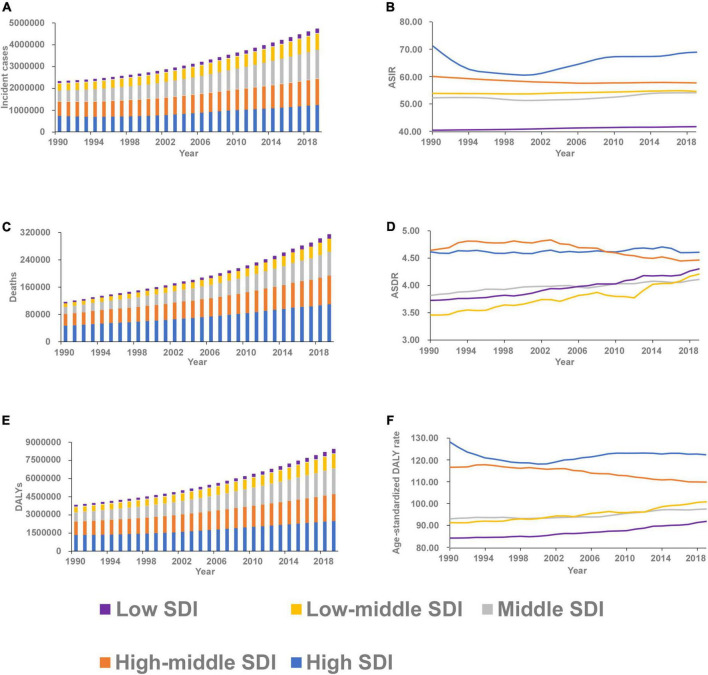
The burden and trend of atrial fibrillation in five socio-demographic Index (SDI) quintiles from 1990 to 2019. **(A)** Incident cases. **(B)** Age-standardized incidence rate (ASIR). **(C)** Deaths. **(D)** Age-standardized death rate (ASDR). **(E)** Disability-adjusted life-years (DALYs). **(F)** Age-standardized DALY rate.

Figure 1 shows that the incident cases of AF increased in all SDI countries from 1990 to 2019, with the smallest increase in high SDI countries (0.67-fold) and the largest increase in middle SDI countries (1.60-fold). All SDI countries also experienced an increase in deaths and DALYs, with the high SDI countries having the highest number in 2019. Compared to other SDI countries, the high SDI countries had the highest ASIR, ASDR and age-standardized DALY rate in 2019. Prior to the early 2000s, ASIR and age-standardized DALY rate in high SDI countries decreased, but then began to increase. However, they increased in middle, low-middle and low SDI countries from 1990 to 2019. Interestingly, the ASIR, ASDR and age-standardized DALY rate decreased in the high-middle SDI countries.

### Regional and national burden of atrial fibrillation

**FIGURE 2:**
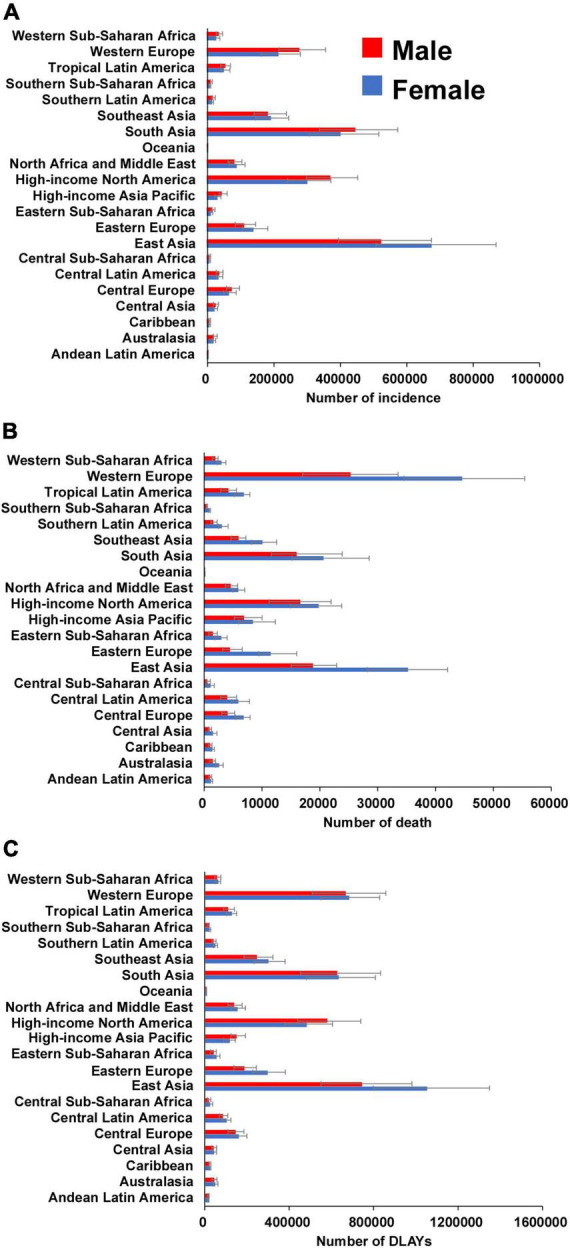
The number of incidence **(A)**, death **(B)**, and DLAYs **(C)** of atrial fibrillation for 21 GBD regions by sex in 2019. Error bars indicate 95% uncertainty intervals. GBD, Global Burden of Diseases; DLAYs, disability-adjusted life-year.

**FIGURE 3:**
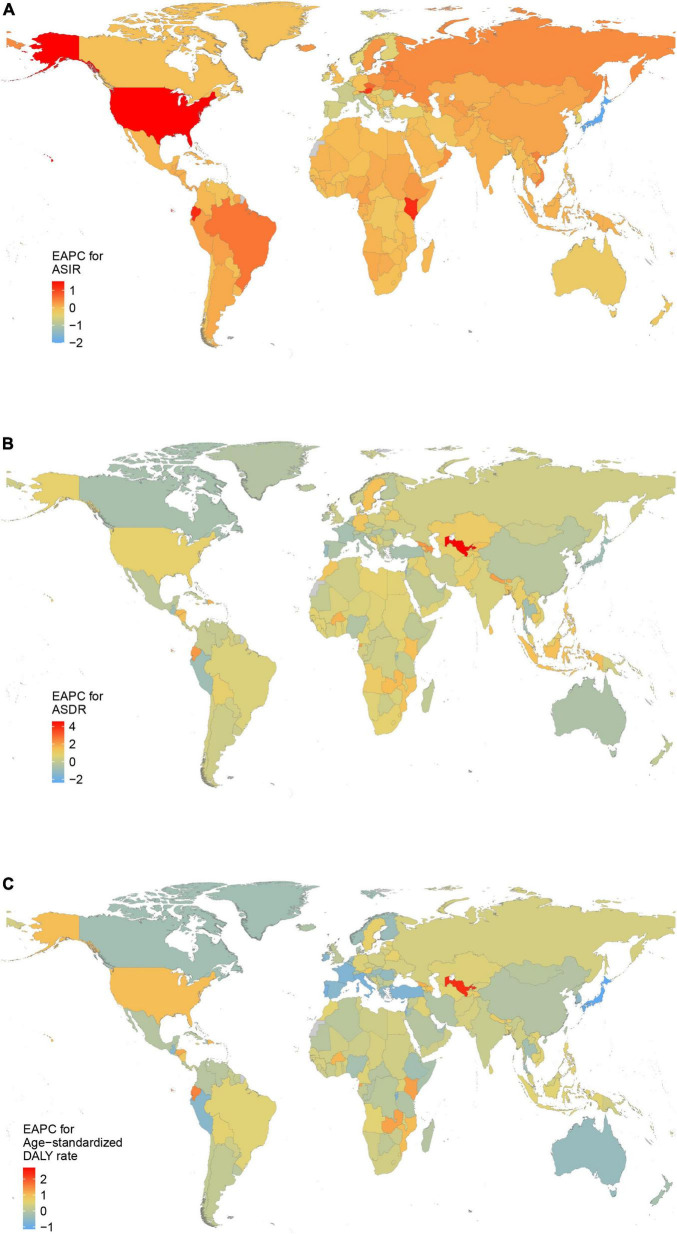
The estimated annual percentage change (EAPC) of atrial fibrillation in 204 countries and territories between 1990 and 2019. **(A)** The EAPC of age-standardized incidence rate (ASIR). **(B)** The EAPC of age- standardized death rate (ASDR). **(C)** The EAPC of age-standardized disability-adjusted life-year (DALY) rate.

Regionally, the highest absolute number of incident cases was estimated in East Asia, with 1,202,906 cases (95% UI 907,838 to 1,545,466) in 2019, while high-income North America contributed the highest ASIR of 108.53 per 100,000 persons (95% UI 87.59–131.44) (Table 1 and Figure 2). From 1990 to 2019, the ASIR of AF increased the most in High-income North America (EAPC 1.33, 95% CI 0.93–1.73) and decreased the most in High-income Asia Pacific (EAPC –1.58, 95% CI –1.92 to –1.23) (Table 1). One in five newly diagnosed cases in 2019 were reported in China (1,165,105, 95% UI 877,577–1,498,665), with India and the United States of America following closely behind (Supplementary Figure 1 and Supplementary Table 1). The highest ASIR was found in the United States of America (109.52 per 100,000 persons, 95% UI 88.97 to 131.92) (Supplementary Figure 2 and Supplementary Table 1). The EAPC of ASIR between 1990 and 2019 was highest in United States of America (1.51, 95% CI 1.05–1.97) and lowest in Japan (–2.01, 95% CI –2.44 to –1.58) (Figure 3).

Most deaths occurred in Western Europe in 2019 (69,928, 95% UI 55,541–84,441), while the highest ASDR was detected in Australasia (6.86 per 100,000 persons, 95% UI 5.45–8.33) (Table 2 and Figure 2). Only in high-income Asia Pacific, Australasia and East Asia, ASDR decreased between 1990 and 2019 (Table 2). There were the most AF-related deaths in 2019 among the countries with the largest populations, such as China, United States of America, and India. The ASDR was highest in Montenegro, Qatar and Bahrain (Supplementary Figures 1, 2 and Supplementary Table 1). The ASDR increased the most in Uzbekistan (EAPC: 4.58; 95% CI 4.21–4.96) and decreased the most in Guam (EAPC: –2.37; 95% CI –2.90 to –1.85) between 1990 and 2019 (Figure 3).

East Asia was the region with the highest DALYs due to AF in 2019 (1,793,188, 95% UI 1,356,400–2,304,890), while Australasia had the highest age-standardized DALY rate (168.28 per 100,000 persons, 95% UI 132.54–214.32) (Table 3 and Figure 2). A notable decline in the age-standardized DALY rate during the study period was found in high-income Asia Pacific, Australasia and Western Europe (Table 3). The highest number of DALYs was detected in China, India, and the United States of America, whereas the Montenegro, Greenland, and Sweden were the three countries with the highest age-standardized DALY rate (Supplementary Figures 1, 2 and Supplementary Table 1). The EAPC of age-standardized DALY rate ranged from –1.14 (95% CI –1.33 to –0.96) in Japan to 2.71 (95% CI 2.25–3.16) in Bahrain (Figure 3).

### Age and sex patterns

**FIGURE 4:**
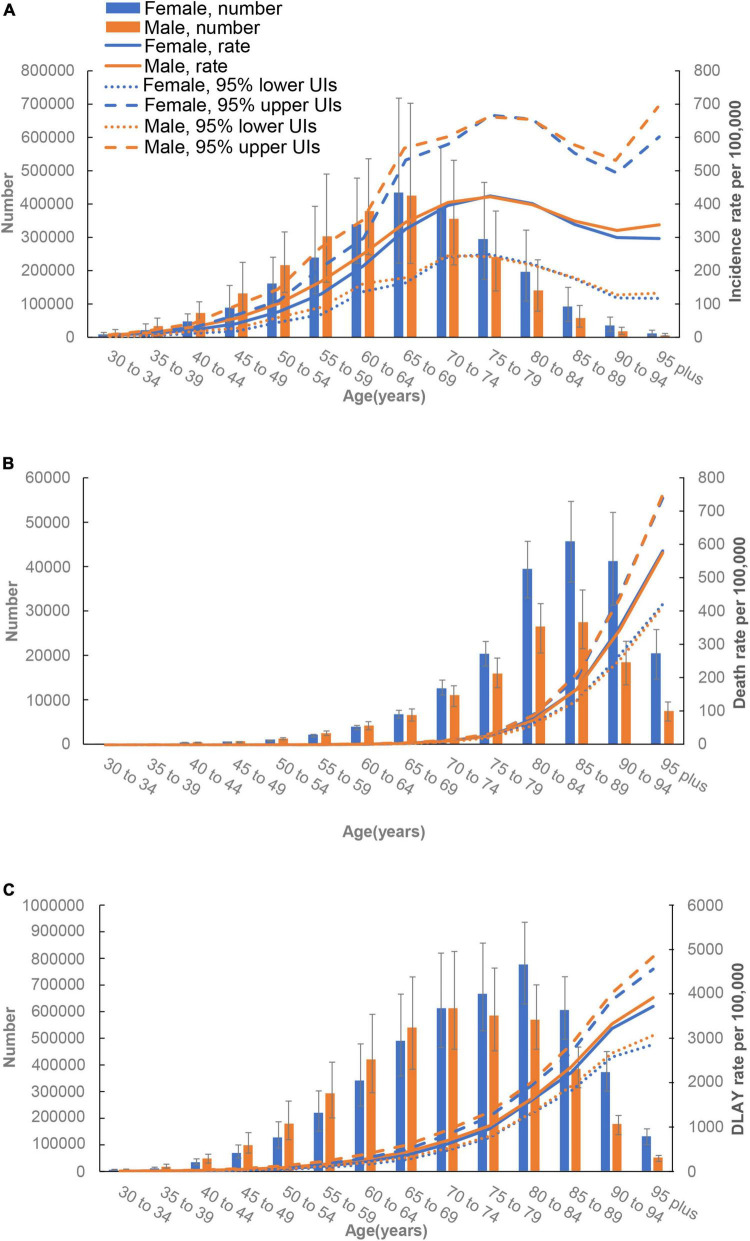
Age-specific numbers and rates of incident cases **(A)**, death cases **(B)**, and disability-adjusted life-years (DALYs) **(C)** of atrial fibrillation by sex, 2019.

The largest incident cases of AF was noted in the 65–69 age groups in both sexes worldwide, while the age-specific incidence rates for both sexes peaked at the 75–79 age groups (Figure 4). The number of deaths peaked at the 75–79 age groups in both sexes, and the global number of DALYs peaked at the 70–74 and 80–84 age groups in males and females, respectively. An increasing trend of death and DALY rates was observed among both sexes, continuing up to the oldest age groups. Additionally, the global DALY rate was lower among females than males across all age groups (Figure 4).

### The correlation between socio-demographic index and global burden of atrial fibrillation

**FIGURE 5:**
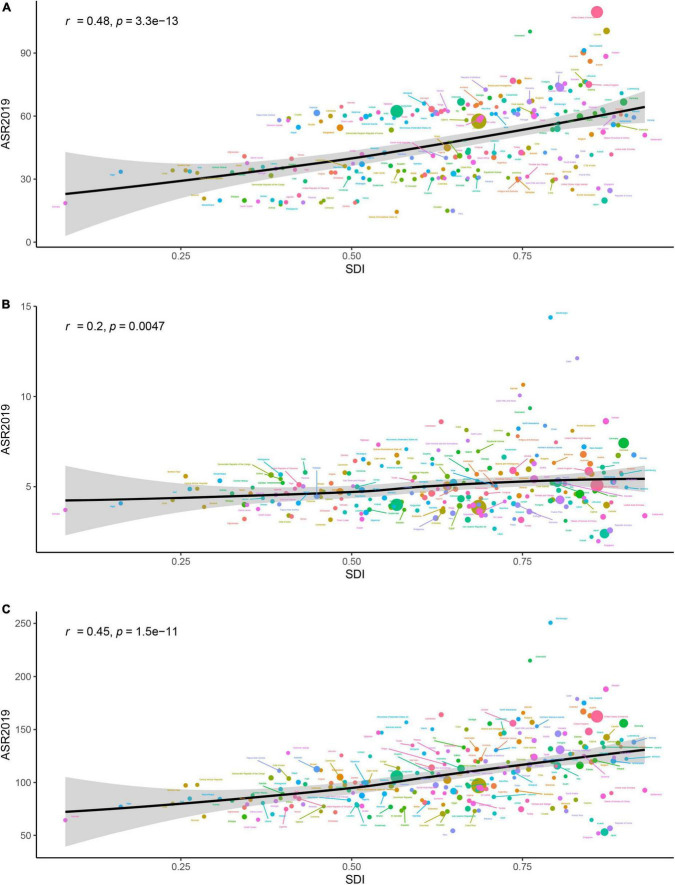
The association between age-standardized incidence rate (ASR) of atrial fibrillation and socio-demographic index (SDI) for 204 countries and territories in 2019. Expected values are shown as the black line. The *r* indices and *P*-value were derived from Pearson correlation analysis. **(A)** Age-standardized incidence rate. **(B)** Age-standardized death rate (ASDR). **(C)** Age-standardized disability-adjusted life-year (DALY) rate.

Our study examined the association between SDI and ASIR, ASDR, and age-standardized DALY rate in 204 countries and territories in 2019 (Figure 5). The results showed that positive correlation occurred between SDI and ASIR, ASDR, and age-standardized DALY rate in 204 countries and territories in 2019 (*P* < 0.01). In particular, a number of countries had much higher ASIR, ASDR, and age-standardized DALY rate than expected based on SDI.

### Attributable burden of atrial fibrillation to risk factors

**FIGURE 6:**
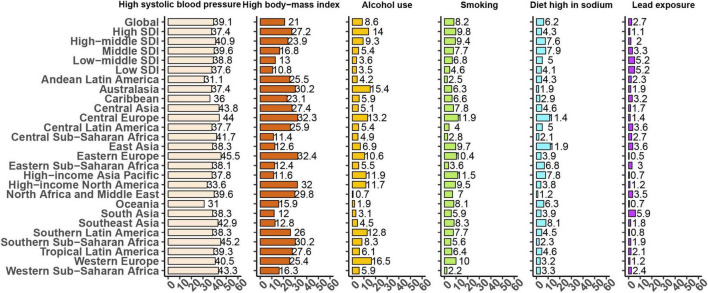
Proportion of age-standardized disability-adjusted life-years (DALYs) due to atrial fibrillation attributable to risk factors for 21 Global Burden of Disease regions, both sexes, 2019.

**FIGURE 7:**
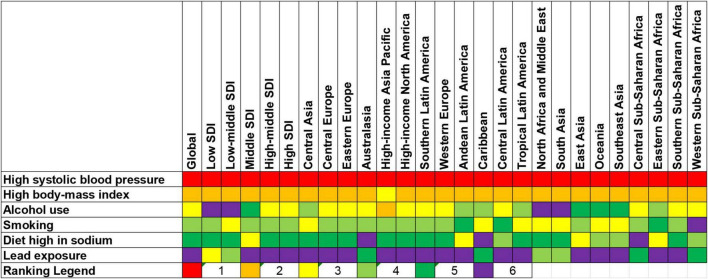
Ranking for all risk factors of atrial fibrillation according to the proportion of age-standardized disability-adjusted life-years (DALYs) rate by locations in 2019. For each risk factor, a higher ranking indicates that it contributed to a higher proportion of age-standardized DALYs rate of atrial fibrillation within a GBD region.

At the global level, the DALYs of AF were primarily attributable to high SBP (39.1%), followed by high BMI (21%), alcohol use (8.6%), smoking (8.2%), diet high in sodium (6.2%), and lead exposure (2.7%) (Figure 6). The percentage of DALYs attributable to these risk factors differed at the region levels (Figures 6, 7). For example, the percentage of DALYs attributable to high SBP was highest in Eastern Europe, while high SBP was still prevalent, and lowest in Oceania. The DALYs attributable to these risk factors also varied by regions for males and females (Supplementary Figures 3, 4).

In addition, the percentage of DALYs attributable to the risk factors also varied across age groups (Supplementary Figure 5). The highest percentage of global DALYs attributable to high SBP was found in the 60–64 age group, while the lowest percentage of global DALYs to high SBP was observed in the 95 plus age group. The estimates of DALYs attributable to these risk factors by age were presented separately for males and females in Appendix (Supplementary Figures 6, 7).

## Discussion

This study comprehensively assessed the incidence, death, and DALY of AF along with the temporal trends over a 30-year period across 204 countries and territories. Globally, there were 4,720,324 incident cases, 117,038 deaths and 8,393,635 DALYs in 2019. The global number of incident cases, deaths and DALYs all increased dramatically between 1990 and 2019, but this may be largely due to population growth and aging since the ASRs have not changed substantially. However, the burden and trend of AF varied in different subgroups by sex, age, SDI levels, regions, and nations.

Our present study differs in some aspects from two previous studies based on the Global Burden of Disease 2017 (5, 6), because of different methodologies and data sources. For example, our present study revealed no change for ASIR between 1990 and 2019, but the ASIR was decreased by 0.15 per year from 39.89 per 100,000 persons to 38.16 per 100,000 persons in the GBD 2017 (6). Furthermore, ASIR, ASDR, and age-standardized DALY rate and their associations with SDI in 2019 were all included in our present study, while previous study has only examined the association of age-standardized DALY rate and SDI (6). In addition, this study also demonstrated that the risk factors for AF varied in different regions and age groups.

Globally, both the ASIR and age-standardized DALY rate were much higher than the ASDR. However, it is not enough to ignore the deaths associated with AF. It is more common for patients to die from comorbidities and complications than from AF alone, because the clinical record typically shows comorbidities and complications as major causes of death (14). In addition, the diagnosis of AF sometimes is difficult, because AF is possibly paroxysmal or asymptomatic onset. The real number of deaths caused by AF may be difficult to identify and quantify. Therefore, the actual global burden of the deaths associated with AF is difficult to estimate, but it is likely greater than generally perceived.

Large geographic heterogeneity in the burden of AF was detected in the present study. High-income North America was the region with the highest ASIR in 2019, while Australasia had the highest ASDR and age-standardized DALY rate. Moreover, all the ASIR, ASDR, and age-standardized DALY rate were positively associated with SDI in 204 countries and territories, which means that countries with higher SDI level experienced higher burden of AF. These findings are generally in agreement with several previous studies (15–17). Differences between countries may due to differences in etiological factors, detection rates, ethnicity, health resource management and medical care. With higher medical care and people’s health awareness, more patients with AF have more possibility to be identified and reported in high SDI regions. Meanwhile, given that the data collection was limited to the regions without sound medical system, the overall burden of AF may be underestimated.

The temporal trend of AF between 1990 and 2019 varied by regions and countries. Interestingly, in high SDI countries, the ASIR began to rise after 2000 partly due to the widespread use of mobile health devices in the past two decades (18). On the contrary, the ASIR in low SDI countries increased throughout the study period. The increased incidence of AF in low SDI countries may be associated with lifestyle westernization (19), widespread screening and an increased life expectancy. Although the ASIR was relatively low in low SDI countries, the age-standardized DALY rate was high enough. AF has created an enormous burden on health systems in low SDI countries, and this situation is expected to deteriorate over the next few decades (20).

As shown in this study, the ASIR and age-standardized DALY rate were significantly higher in males than in females, while the ASDR was higher in females than in males. This gender disparity of the burden of AF is similar to previous studies (21–23). Females and males had distinct differences in symptom severity, mechanisms and etiology, response to treatment, and outcomes (21, 22). Advancing our knowledge about the risk and benefit of different management strategies may reduce the public health burden of AF.

Our results demonstrated that a peak in mortality and morbidity occurred in the elderly. Previous studies have reported that the majority of patients were diagnosed with AF at ≥60 years, and the incidence of AF increased with age (2, 24–27). In addition, the risks of cardiovascular death and stroke/systemic embolism were higher among elderly with AF (28). Taking Oral anticoagulation can decrease the risk of ischemic stroke and all-cause mortality. However, increased risk of bleeding during antithrombotic therapy was found with increasing age (29), resulting in challenges in management, such as anticoagulation monitoring and medication compliance, as well as the presence of comorbidities. Although elderly patients need careful treatment decisions because of biological changes and specific disease management aspects (29), they were under-represented by major cardiovascular trials and secondary cardiovascular preventive and rehabilitation programs. In the context of accelerated global aging, more researches about the evidence-based care of the elderly with AF are required.

Based on the GBD study, this study analyzed six modifiable risk factors attributed to the DALYs of AF, including high SBP, high BMI, alcohol use, smoking, diet high in sodium and lead exposure. This emphasized the main role of risk factor modification in alleviating the burden of AF. The association between AF and high SBP, high BMI, alcohol use, smoking and diet high in sodium has long been recognized (30, 31). Although the relationship between lead exposure and abnormal ECG conduction has rarely been reported (32), lead exposure contributing to hypertension and cardiovascular diseases has been known and recognized widely (33). Given the fact that hypertension and cardiovascular diseases increase the risk of AF, a tight relationship exists between AF and lead exposure. More importantly, the current study indicated that the percentage of DALYs attributable to risk factors also varied by regions, gender and age groups. No matter what approach is taken to mitigate these risk factors, it is critical to tailor interventions to the context of the target audience.

Several limitations need to be mentioned in this study. Firstly, some countries struggled with data quality, and especially data were missing in several countries. The burden of AF in these countries were estimated by the GBD modeling process. Higher quality data is still needed to monitor the burden of AF and its risk factors, as well as evaluate the effectiveness of population-based interventions. Secondly, the estimates of AF in the GBD study were not stratified based on the different clinical subtypes. Thirdly, other risk factors, such as diabetes, low physical activity and hyperthyroidism have not yet been included in GBD study. Finally, the outbreak of COVID-19 virus significantly affects the burden of AF (34), more data are needed to identify the burden and trend of AF during and after the epidemic.

## Conclusion

AF remains a major public health challenge with large geographic variations in burden. There was a positive association between AF and the SDI of each country and territory. Additionally, the burden was higher in males and elderly. More strategies aimed at implementing effective and geo-specific interventions and addressing modifiable risk factors are needed to counteract and mitigate the future burden of AF.

## Data availability statement

The datasets presented in this study can be found in online repositories. The names of the repository/repositories and accession number(s) can be found below: http://ghdx.healthdata.org/gbd-results-tool.

## Ethics statement

Ethical review and approval was not required for this study in accordance with the local legislation and institutional requirements. Written informed consent was not required for this study in accordance with the local legislation and institutional requirements.

## Author contributions

SX, YC, and MX contributed to the conception and design of the study. SX, RL, WH, and HZ performed the statistical analysis. RL, WH, and YL wrote the first draft of the manuscript. SX and MX reviewed and edited the manuscript. All authors contributed to the article and approved the final manuscript.

## Footnotes

1
http://ghdx.healthdata.org/gbd-results-tool

## Supplementary material

Supplementary Figure 1 The number of incident cases **(A)**, death cases **(B)**, and disability-adjusted life-years (DALYs) **(C)** of atrial fibrillation in 204 countries and territories in 2019.

Supplementary Figure 2 The age-standardized rates of atrial fibrillation in 204 countries and territories in 2019. **(A)** Age-standardized incidence rate. **(B)** Age-standardized death rate (ASDR). **(C)** Age-standardized disability-adjusted life-year (DALY) rate.

Supplementary Figure 3 Percentage of disability-adjusted life-years (DALYs) due to atrial fibrillation attributable to risk factors for 21 Global Burden of Disease regions, 2019, males.

Supplementary Figure 4 Percentage of disability-adjusted life-years (DALYs) due to atrial fibrillation attributable to risk factors for 21 Global Burden of Disease regions, 2019, females.

Supplementary Figure 5 Percentage of disability-adjusted life-years (DALYs) due to atrial fibrillation attributable to risk factors by age for both sexes, 2019.

Supplementary Figure 6 Percentage of disability-adjusted life-years (DALYs) due to atrial fibrillation attributable to risk factors by age for males, 2019.

Supplementary Figure 7 Percentage of disability-adjusted life-years (DALYs) due to atrial fibrillation attributable to risk factors by age for females, 2019.
