# Supplementary material for: Burden of atrial fibrillation and its attributable risk factors from 1990 to 2019: An analysis of the Global Burden of Disease study 2019
Source: Front Cardiovasc Med. 2022 Oct 26;9:997698. doi: 10.3389/fcvm.2022.997698 (PMC9643162; doi:10.3389/fcvm.2022.997698)
Supplement: Supplementary file 1 [file Table_1.docx]

**Table s1. Incidence, deaths and DALYs for atrial fibrillation in 2019, and temporal trends from 1990 to 2019 in 204 countries and territories.**

| **Location** | **Incidence** | | | **Death** | | | **DALYs** | | |
| --- | --- | --- | --- | --- | --- | --- | --- | --- | --- |
|  | **Count (95%UI)** | **ASIR per 100,000 (95% UI)** | **EAPC (95% CI)** | **Count (95% UI)** | **ASDR per 100,000 (95% UI)** | **EAPC (95% CI)** | **Count (95% UI)** | **Age-standardized DALY rates per 100,000 (95% UI)** | **EAPC (95% CI)** |
| Afghanistan | 4586  (3474 to 5854) | 40.85  (30.96 to 52.69) | 0.15  (0.13 to 0.17) | 216  (159 to 306) | 3.20  (2.30 to 4.23) | 0.68  (0.62 to 0.74) | 7079  (5364 to 9386) | 76.41  (59.48 to 98.79) | 0.37  (0.34 to 0.40) |
| Albania | 2784  (2095 to 3613) | 64.84  (49.47 to 83.47) | 0.04  (0.01 to 0.06) | 157  (119 to 198) | 3.97  (3.01 to 4.98) | 0.23  (0.14 to 0.31) | 5202  (3928 to 6861) | 120.14  (91.29 to 157.68) | 0.14  (0.10 to 0.18) |
| Algeria | 13207  (10042 to 17165) | 40.50  (30.55 to 52.60) | 0.00  (-0.01 to 0.01) | 967  (740 to 1230) | 4.87  (3.67 to 6.11) | 0.23  (0.17 to 0.29) | 24352  (18956 to 30290) | 91.18  (71.78 to 111.62) | 0.03  (0.00 to 0.06) |
| American Samoa | 30  (23 to 39) | 64.33  (48.79 to 82.78) | 0.08  (0.04 to 0.12) | 2  (1 to 2) | 4.92  (3.92 to 6.14) | 0.14  (0.07 to 0.21) | 56  (44 to 69) | 131.02  (104.81 to 162.47) | 0.11  (0.09 to 0.13) |
| Andorra | 84  (63 to 107) | 60.69  (45.67 to 77.75) | -0.71  (-0.80 to -0.63) | 10  (7 to 13) | 5.78  (3.83 to 7.76) | 0.17  (0.04 to 0.30) | 197  (146 to 259) | 133.72  (99.85 to 176.65) | -0.32  (-0.39 to -0.26) |
| Angola | 3500  (2660 to 4513) | 33.15  (25.02 to 42.47) | 0.05  (0.03 to 0.06) | 290  (206 to 367) | 5.19  (3.66 to 6.61) | 1.03  (1.00 to 1.06) | 7878  (5868 to 9853) | 99.37  (74.90 to 123.73) | 0.56  (0.54 to 0.59) |
| Antigua and Barbuda | 29  (22 to 37) | 29.31  (21.97 to 37.80) | 0.14  (0.13 to 0.15) | 6  (5 to 8) | 7.80  (6.45 to 9.95) | 0.85  (0.70 to 1.01) | 100  (84 to 121) | 114.01  (96.92 to 139.06) | 0.47  (0.35 to 0.60) |
| Argentina | 21664  (16367 to 27880) | 40.34  (30.69 to 51.65) | 0.13  (0.10 to 0.16) | 2906  (2405 to 3730) | 5.14  (4.25 to 6.59) | 0.21  (0.06 to 0.36) | 53856  (44272 to 66837) | 96.73  (79.44 to 120.36) | 0.14  (0.08 to 0.20) |
| Armenia | 2801  (2117 to 3617) | 66.14  (50.45 to 84.61) | 0.09  (0.08 to 0.10) | 162  (132 to 219) | 4.35  (3.53 to 5.98) | 1.28  (1.11 to 1.45) | 4990  (3809 to 6604) | 122.00  (93.57 to 160.53) | 0.51  (0.45 to 0.59) |
| Australia | 35779  (27035 to 46213) | 90.24  (68.96 to 115.19) | -0.25  (-0.30 to -0.20) | 3423  (2740 to 4172) | 6.79  (5.41 to 8.24) | -0.51  (-0.58 to -0.44) | 75139  (59551 to 95423) | 167.01  (131.41 to 213.42) | -0.40  (-0.43 to -0.37) |
| Austria | 13490  (10556 to 16980) | 86.10  (67.28 to 108.91) | 1.04  (0.75 to 1.32) | 1453  (1133 to 1655) | 6.27  (4.89 to 7.12) | 0.29  (0.06 to 0.51) | 31779  (24891 to 40121) | 163.04  (126.86 to 207.93) | 0.78  (0.53 to 1.04) |
| Azerbaijan | 6702  (5031 to 8764) | 65.25  (49.70 to 84.05) | 0.15  (0.12 to 0.18) | 226  (189 to 262) | 5.69  (4.57 to 6.67) | 2.18  (1.92 to 2.46) | 9149  (6883 to 12077) | 131.39  (103.70 to 165.30) | 0.95  (0.85 to 1.06) |
| Bahamas | 112  (84 to 144) | 29.84  (22.22 to 38.81) | -0.02  (-0.03 to 0.00) | 19  (15 to 25) | 6.32  (5.06 to 8.22) | 0.30  (0.09 to 0.52) | 355  (293 to 441) | 104.43  (86.30 to 129.47) | 0.19  (0.07 to 0.31) |
| Bahrain | 433  (325 to 565) | 42.26  (31.85 to 54.76) | -0.13  (-0.13 to -0.12) | 35  (26 to 44) | 10.65  (8.28 to 12.78) | 4.50  (3.74 to 5.25) | 946  (717 to 1174) | 165.78  (129.43 to 198.77) | 2.71  (2.25 to 3.16) |
| Bangladesh | 70657  (53211 to 90845) | 54.48  (41.11 to 69.99) | -0.10  (-0.11 to -0.08) | 4164  (2642 to 5589) | 4.73  (2.93 to 6.37) | 1.47  (1.18 to 1.75) | 118576  (88486 to 151181) | 105.11  (78.77 to 133.15) | 0.55  (0.44 to 0.66) |
| Barbados | 151  (111 to 198) | 30.33  (22.71 to 39.56) | 0.11  (0.10 to 0.11) | 28  (22 to 35) | 5.71  (4.59 to 7.19) | 0.46  (0.37 to 0.56) | 456  (376 to 561) | 92.33  (76.17 to 113.36) | 0.26  (0.20 to 0.32) |
| Belarus | 12004  (9053 to 15426) | 76.34  (57.97 to 97.49) | 0.38  (0.37 to 0.40) | 752  (586 to 965) | 4.50  (3.51 to 5.78) | 1.03  (0.93 to 1.12) | 22656  (16908 to 29587) | 138.54  (103.48 to 181.55) | 0.64  (0.59 to 0.70) |
| Belgium | 10191  (7912 to 13076) | 51.25  (39.90 to 65.15) | -0.67  (-0.82 to -0.51) | 1770  (1431 to 2231) | 5.71  (4.61 to 7.20) | -0.20  (-0.27 to -0.14) | 30619  (25209 to 37943) | 119.42  (96.46 to 149.63) | -0.40  (-0.46 to -0.34) |
| Belize | 82  (63 to 105) | 31.41  (23.67 to 40.94) | 0.21  (0.19 to 0.23) | 10  (8 to 13) | 4.53  (3.56 to 5.89) | 0.58  (0.14 to 1.02) | 201  (167 to 247) | 83.32  (69.27 to 101.13) | 0.40  (0.12 to 0.69) |
| Benin | 1612  (1234 to 2073) | 34.37  (25.96 to 44.55) | 0.04  (0.03 to 0.05) | 131  (103 to 162) | 4.63  (3.65 to 5.68) | 0.73  (0.61 to 0.86) | 3063  (2442 to 3764) | 83.71  (67.66 to 102.29) | 0.40  (0.33 to 0.47) |
| Bermuda | 40  (30 to 53) | 30.93  (23.24 to 39.99) | -0.14  (-0.17 to -0.11) | 7  (5 to 9) | 4.69  (3.67 to 6.00) | -0.93  (-1.01 to -0.83) | 108  (88 to 134) | 76.91  (62.35 to 95.72) | -0.90  (-0.96 to -0.83) |
| Bhutan | 320  (244 to 411) | 57.47  (43.71 to 73.71) | -0.09  (-0.11 to -0.07) | 19  (13 to 27) | 4.79  (3.15 to 6.75) | 1.78  (1.70 to 1.85) | 539  (403 to 691) | 111.59  (83.06 to 143.06) | 0.73  (0.70 to 0.75) |
| Bolivia  (Plurinational State of) | 1175  (881 to 1532) | 14.41  (10.71 to 18.68) | 0.20  (0.17 to 0.24) | 419  (309 to 560) | 6.75  (4.95 to 8.92) | 0.87  (0.84 to 0.90) | 6739  (5107 to 8988) | 92.97  (70.89 to 121.81) | 0.60  (0.57 to 0.62) |
| Bosnia and Herzegovina | 4136  (3119 to 5310) | 69.58  (53.44 to 88.80) | -0.09  (-0.11 to -0.07) | 295  (232 to 388) | 5.75  (4.53 to 7.48) | 1.11  (0.64 to 1.57) | 8538  (6614 to 10978) | 145.72  (113.24 to 186.08) | 0.36  (0.20 to 0.51) |
| Botswana | 461  (351 to 595) | 34.70  (26.26 to 44.71) | 0.10  (0.09 to 0.11) | 28  (20 to 37) | 3.80  (2.71 to 5.21) | 0.19  (-0.06 to 0.44) | 817  (625 to 1036) | 79.07  (60.67 to 100.19) | 0.10  (-0.07 to 0.27) |
| Brazil | 105682  (79889 to 136209) | 44.91  (33.89 to 58.00) | 0.56  (0.39 to 0.73) | 10811  (8637 to 12801) | 5.04  (4.01 to 5.97) | 0.52  (0.32 to 0.72) | 230116  (189167 to 279886) | 102.50  (84.33 to 124.48) | 0.52  (0.39 to 0.66) |
| Brunei Darussalam | 90  (68 to 115) | 24.96  (19.10 to 32.13) | -0.33  (-0.37 to -0.29) | 10  (9 to 12) | 8.10  (6.99 to 9.22) | 0.36  (0.19 to 0.54) | 265  (224 to 315) | 127.10  (110.49 to 146.41) | -0.01  (-0.09 to 0.08) |
| Bulgaria | 8975  (6718 to 11599) | 67.26  (51.18 to 85.95) | -0.13  (-0.16 to -0.10) | 931  (720 to 1448) | 6.41  (4.99 to 9.99) | 0.27  (0.08 to 0.46) | 23545  (18078 to 31008) | 156.83  (120.82 to 204.60) | 0.17  (0.10 to 0.24) |
| Burkina Faso | 2994  (2277 to 3866) | 34.13  (25.88 to 43.82) | -0.09  (-0.11 to -0.07) | 287  (210 to 381) | 5.58  (4.09 to 7.44) | 1.71  (1.50 to 1.94) | 6470  (4941 to 8115) | 97.29  (75.09 to 121.70) | 1.09  (0.96 to 1.22) |
| Burundi | 876  (665 to 1142) | 20.74  (15.57 to 27.18) | 0.06  (0.04 to 0.08) | 93  (56 to 128) | 3.87  (2.28 to 5.24) | -0.86  (-0.98 to -0.73) | 2222  (1553 to 2911) | 67.89  (46.25 to 88.39) | -0.83  (-0.94 to -0.71) |
| Cabo Verde | 148  (114 to 188) | 35.33  (26.91 to 45.13) | 0.02  (0.01 to 0.04) | 27  (21 to 33) | 6.29  (4.87 to 7.70) | 1.67  (1.42 to 1.93) | 414  (336 to 496) | 100.80  (81.80 to 121.10) | 0.88  (0.72 to 1.04) |
| Cambodia | 6978  (5215 to 8924) | 60.03  (44.84 to 76.84) | 0.17  (0.12 to 0.23) | 232  (184 to 290) | 3.16  (2.47 to 3.94) | 1.18  (1.12 to 1.23) | 9447  (7052 to 12396) | 95.56  (72.43 to 124.50) | 0.52  (0.51 to 0.54) |
| Cameroon | 4153  (3159 to 5334) | 36.19  (27.39 to 46.64) | 0.20  (0.16 to 0.23) | 350  (274 to 439) | 5.78  (4.46 to 7.24) | 0.65  (0.61 to 0.69) | 8128  (6452 to 9994) | 97.63  (78.11 to 119.12) | 0.49  (0.46 to 0.52) |
| Canada | 67761  (50626 to 87212) | 100.53  (76.24 to 128.26) | -0.05  (-0.06 to -0.04) | 3228  (2505 to 3892) | 3.97  (3.09 to 4.84) | -0.67  (-0.80 to -0.54) | 103817  (78062 to 136908) | 142.69  (106.55 to 188.65) | -0.26  (-0.30 to -0.21) |
| Central African Republic | 653  (492 to 840) | 32.95  (24.91 to 42.20) | -0.08  (-0.09 to -0.07) | 50  (30 to 70) | 4.89  (2.94 to 6.68) | 0.12  (0.06 to 0.19) | 1465  (1043 to 1934) | 97.78  (69.10 to 127.48) | 0.00  (-0.04 to 0.05) |
| Chad | 1831  (1408 to 2353) | 34.15  (25.95 to 43.76) | 0.11  (0.10 to 0.11) | 132  (95 to 176) | 4.25  (3.07 to 5.63) | 0.57  (0.51 to 0.64) | 3320  (2538 to 4230) | 79.93  (61.33 to 100.20) | 0.39  (0.35 to 0.43) |
| Chile | 10808  (8123 to 13901) | 44.84  (33.73 to 57.62) | -0.08  (-0.38 to 0.22) | 1411  (1165 to 2052) | 5.97  (4.91 to 8.69) | 0.33  (0.21 to 0.45) | 27026  (22203 to 33915) | 112.55  (92.57 to 141.10) | 0.17  (0.07 to 0.28) |
| China | 1165105  (877577 to 1498665) | 57.57  (43.76 to 73.12) | 0.17  (0.04 to 0.29) | 51748  (43605 to 60138) | 3.84  (3.20 to 4.45) | -0.28  (-0.34 to -0.22) | 1729840  (1309686 to 2226445) | 97.08  (75.21 to 123.12) | -0.05  (-0.14 to 0.04) |
| Colombia | 16343  (12346 to 21106) | 30.85  (23.31 to 39.92) | 0.01  (-0.01 to 0.04) | 2128  (1528 to 2834) | 3.64  (2.64 to 4.76) | 0.05  (-0.10 to 0.21) | 38546  (30514 to 48095) | 70.54  (55.84 to 88.37) | -0.01  (-0.09 to 0.06) |
| Comoros | 88  (67 to 112) | 18.79  (14.27 to 24.30) | -0.03  (-0.04 to -0.03) | 16  (11 to 20) | 4.43  (3.04 to 5.71) | -0.04  (-0.15 to 0.07) | 302  (226 to 372) | 72.70  (54.24 to 89.34) | -0.16  (-0.26 to -0.06) |
| Congo | 844  (640 to 1094) | 33.79  (25.47 to 43.79) | 0.02  (0.01 to 0.03) | 88  (68 to 114) | 6.34  (4.75 to 8.20) | 0.14  (0.03 to 0.25) | 2164  (1682 to 2658) | 113.56  (90.29 to 139.80) | -0.04  (-0.13 to 0.05) |
| Cook Islands | 15  (11 to 20) | 61.55  (46.52 to 79.23) | 0.10  (0.08 to 0.13) | 1  (1 to 1) | 4.79  (3.85 to 5.82) | 0.00  (-0.10 to 0.09) | 30  (24 to 38) | 124.97  (99.65 to 157.03) | -0.08  (-0.14 to -0.03) |
| Costa Rica | 1609  (1219 to 2064) | 31.48  (23.68 to 40.64) | 0.00  (-0.01 to 0.00) | 204  (144 to 260) | 3.82  (2.69 to 4.88) | 0.10  (-0.12 to 0.33) | 3733  (2930 to 4709) | 73.08  (57.40 to 92.39) | 0.06  (-0.06 to 0.17) |
| Croatia | 4686  (3642 to 5811) | 58.91  (46.39 to 73.03) | -0.20  (-0.63 to 0.23) | 319  (250 to 439) | 3.39  (2.65 to 4.62) | -0.18  (-0.29 to -0.06) | 9496  (7231 to 12316) | 103.77  (78.57 to 135.05) | -0.27  (-0.53 to -0.02) |
| Cuba | 5591  (4155 to 7204) | 29.18  (21.79 to 37.55) | 0.05  (0.03 to 0.06) | 926  (716 to 1222) | 4.27  (3.32 to 5.68) | 0.04  (-0.13 to 0.21) | 15261  (12276 to 18913) | 75.58  (60.87 to 94.11) | 0.01  (-0.11 to 0.12) |
| Cyprus | 1121  (847 to 1443) | 58.04  (44.11 to 74.57) | -0.70  (-0.93 to -0.47) | 127  (108 to 144) | 8.14  (6.79 to 9.25) | -0.71  (-0.86 to -0.56) | 2775  (2280 to 3420) | 150.07  (124.21 to 182.95) | -0.89  (-0.99 to -0.78) |
| Czechia | 14575  (10946 to 18847) | 76.39  (58.51 to 97.37) | 0.67  (0.42 to 0.92) | 940  (719 to 1145) | 4.17  (3.17 to 5.09) | -0.08  (-0.15 to 0.00) | 29179  (22356 to 38060) | 133.84  (102.33 to 174.44) | 0.33  (0.19 to 0.48) |
| Côte d'Ivoire | 3650  (2782 to 4717) | 35.74  (27.08 to 46.23) | -0.01  (-0.03 to 0.00) | 226  (179 to 281) | 4.59  (3.52 to 5.73) | 0.39  (0.33 to 0.45) | 5979  (4698 to 7555) | 83.68  (66.98 to 103.23) | 0.21  (0.18 to 0.24) |
| Democratic People's Republic of Korea | 16971  (12779 to 21671) | 53.38  (40.40 to 68.62) | 0.01  (0.00 to 0.02) | 919  (732 to 1113) | 3.85  (3.02 to 4.74) | -0.12  (-0.29 to 0.04) | 28155  (21699 to 35964) | 96.57  (75.12 to 122.93) | -0.05  (-0.11 to 0.02) |
| Democratic Republic of the Congo | 10684  (8148 to 13804) | 31.69  (24.09 to 40.86) | -0.17  (-0.19 to -0.15) | 1156  (678 to 1791) | 5.65  (3.27 to 8.81) | 0.25  (0.13 to 0.37) | 27824  (18995 to 38915) | 104.42  (70.43 to 145.61) | 0.06  (-0.03 to 0.16) |
| Denmark | 5795  (4350 to 7550) | 55.71  (42.39 to 71.53) | -0.52  (-0.57 to -0.47) | 907  (729 to 1072) | 6.73  (5.42 to 7.98) | 0.37  (0.17 to 0.57) | 16963  (13877 to 20897) | 137.23  (111.86 to 170.64) | -0.13  (-0.25 to 0.00) |
| Djibouti | 111  (84 to 143) | 19.78  (15.01 to 25.62) | 0.22  (0.20 to 0.25) | 13  (8 to 18) | 4.73  (2.88 to 6.36) | 0.15  (0.04 to 0.27) | 313  (219 to 414) | 77.52  (53.58 to 100.61) | 0.05  (-0.04 to 0.14) |
| Dominica | 28  (20 to 36) | 30.60  (22.83 to 39.80) | 0.15  (0.14 to 0.16) | 7  (6 to 9) | 7.90  (6.54 to 9.56) | 0.53  (0.43 to 0.63) | 109  (92 to 131) | 119.33  (100.15 to 142.62) | 0.44  (0.35 to 0.52) |
| Dominican Republic | 2708  (2046 to 3495) | 29.99  (22.52 to 38.96) | 0.08  (0.06 to 0.09) | 437  (343 to 540) | 5.52  (4.32 to 6.81) | 1.69  (1.39 to 1.99) | 7736  (6211 to 9458) | 90.95  (73.12 to 111.08) | 1.07  (0.91 to 1.23) |
| Ecuador | 2162  (1643 to 2732) | 14.95  (11.36 to 18.82) | 1.19  (0.92 to 1.44) | 644  (516 to 792) | 5.57  (4.47 to 6.82) | 2.13  (1.78 to 2.49) | 9551  (7759 to 11645) | 72.69  (59.44 to 88.27) | 1.54  (1.25 to 1.83) |
| Egypt | 24805  (18788 to 31731) | 41.12  (30.92 to 52.81) | 0.02  (0.01 to 0.03) | 1311  (853 to 1800) | 3.93  (2.51 to 5.38) | 0.68  (0.48 to 0.88) | 40893  (29899 to 53997) | 85.85  (62.32 to 110.67) | 0.38  (0.29 to 0.47) |
| El Salvador | 1768  (1317 to 2276) | 29.64  (22.00 to 38.29) | 0.09  (0.06 to 0.11) | 294  (219 to 364) | 4.14  (3.09 to 5.14) | 0.36  (0.25 to 0.47) | 4685  (3712 to 5708) | 73.52  (58.16 to 90.41) | 0.21  (0.15 to 0.28) |
| Equatorial Guinea | 158  (122 to 202) | 34.23  (25.90 to 44.28) | 0.22  (0.19 to 0.25) | 20  (12 to 29) | 6.96  (4.49 to 10.58) | 2.05  (1.97 to 2.13) | 429  (304 to 581) | 119.46  (85.19 to 163.85) | 1.25  (1.19 to 1.30) |
| Eritrea | 435  (330 to 563) | 17.91  (13.60 to 23.12) | 0.03  (0.02 to 0.03) | 66  (41 to 89) | 5.43  (3.40 to 7.54) | 0.66  (0.50 to 0.82) | 1505  (1061 to 1947) | 85.11  (59.17 to 111.05) | 0.32  (0.20 to 0.43) |
| Estonia | 1724  (1298 to 2210) | 74.70  (56.79 to 95.71) | 0.47  (0.43 to 0.52) | 149  (116 to 210) | 4.58  (3.59 to 6.43) | 0.77  (0.68 to 0.87) | 3911  (2963 to 5111) | 137.68  (103.30 to 179.54) | 0.56  (0.49 to 0.63) |
| Eswatini | 189  (143 to 244) | 33.97  (25.65 to 43.73) | -0.06  (-0.13 to 0.01) | 11  (8 to 14) | 3.71  (2.67 to 4.89) | 0.98  (0.72 to 1.24) | 332  (257 to 425) | 76.11  (59.09 to 96.47) | 0.46  (0.28 to 0.64) |
| Ethiopia | 7764  (5968 to 9919) | 19.84  (15.06 to 25.55) | 0.26  (0.24 to 0.28) | 1047  (613 to 1377) | 4.00  (2.31 to 5.29) | -0.12  (-0.30 to 0.05) | 21970  (15023 to 27863) | 67.64  (45.72 to 85.46) | -0.25  (-0.39 to -0.11) |
| Fiji | 466  (350 to 601) | 63.54  (48.42 to 80.64) | 0.13  (0.11 to 0.15) | 23  (18 to 28) | 5.22  (4.30 to 6.32) | 0.63  (0.53 to 0.73) | 815  (643 to 1021) | 133.13  (107.11 to 164.82) | 0.40  (0.34 to 0.46) |
| Finland | 6554  (4892 to 8544) | 61.32  (46.32 to 78.87) | -0.48  (-0.53 to -0.43) | 750  (484 to 865) | 4.77  (3.08 to 5.48) | -0.22  (-0.31 to -0.13) | 16183  (12363 to 20604) | 120.88  (92.01 to 154.01) | -0.35  (-0.36 to -0.33) |
| France | 69663  (52720 to 90358) | 59.79  (44.98 to 76.73) | -0.75  (-0.80 to -0.68) | 9253  (6808 to 10773) | 4.59  (3.44 to 5.39) | -0.69  (-0.74 to -0.63) | 179825  (141222 to 226712) | 116.24  (90.34 to 148.61) | -0.68  (-0.71 to -0.65) |
| Gabon | 345  (262 to 443) | 34.02  (25.90 to 43.85) | 0.06  (0.04 to 0.07) | 47  (33 to 62) | 6.92  (4.87 to 9.21) | 0.33  (0.28 to 0.39) | 999  (743 to 1271) | 120.62  (90.40 to 152.56) | 0.15  (0.11 to 0.20) |
| Gambia | 326  (252 to 415) | 35.20  (26.61 to 44.93) | -0.02  (-0.04 to 0.00) | 31  (25 to 39) | 5.21  (4.06 to 6.57) | 1.05  (0.99 to 1.11) | 675  (540 to 833) | 89.47  (71.89 to 109.22) | 0.60  (0.55 to 0.65) |
| Georgia | 3997  (3040 to 5119) | 68.62  (52.43 to 87.96) | 0.11  (0.09 to 0.13) | 421  (332 to 505) | 6.03  (4.74 to 7.17) | 2.07  (1.48 to 2.67) | 9930  (7925 to 12306) | 155.57  (122.96 to 193.37) | 1.14  (0.89 to 1.39) |
| Germany | 108145  (81682 to 138961) | 66.69  (50.48 to 85.63) | -0.08  (-0.27 to 0.10) | 18196  (14793 to 22483) | 7.42  (6.07 to 9.21) | 1.26  (0.95 to 1.56) | 333196  (273203 to 414843) | 155.89  (126.49 to 196.40) | 0.48  (0.29 to 0.67) |
| Ghana | 5667  (4307 to 7279) | 36.33  (27.54 to 46.76) | -0.01  (-0.07 to 0.05) | 330  (271 to 404) | 3.93  (3.19 to 4.77) | 0.68  (0.64 to 0.73) | 9080  (7102 to 11399) | 76.96  (61.54 to 94.77) | 0.38  (0.33 to 0.42) |
| Greece | 10542  (7967 to 13396) | 53.61  (40.50 to 68.25) | -0.61  (-0.66 to -0.56) | 1742  (1360 to 3020) | 5.23  (4.12 to 8.96) | -0.32  (-0.40 to -0.23) | 32485  (25371 to 44510) | 119.96  (92.52 to 160.23) | -0.51  (-0.56 to -0.46) |
| Greenland | 69  (52 to 88) | 100.25  (76.51 to 126.96) | -0.08  (-0.09 to -0.08) | 4  (4 to 5) | 9.35  (7.48 to 10.94) | -0.39  (-0.55 to -0.22) | 128  (103 to 157) | 214.86  (175.09 to 264.43) | -0.26  (-0.35 to -0.17) |
| Grenada | 32  (24 to 42) | 29.82  (22.55 to 39.12) | 0.10  (0.09 to 0.10) | 5  (4 to 6) | 5.94  (5.01 to 7.87) | 0.22  (0.05 to 0.38) | 94  (80 to 116) | 98.21  (83.59 to 121.68) | 0.10  (-0.01 to 0.21) |
| Guam | 118  (90 to 153) | 62.26  (47.00 to 79.85) | 0.25  (0.22 to 0.27) | 6  (5 to 7) | 3.10  (2.52 to 3.72) | -2.37  (-2.90 to -1.85) | 196  (154 to 251) | 105.84  (83.07 to 136.07) | -0.83  (-1.05 to -0.60) |
| Guatemala | 3188  (2394 to 4173) | 29.70  (22.03 to 38.57) | 0.10  (0.06 to 0.14) | 286  (211 to 353) | 3.72  (2.74 to 4.49) | -0.88  (-1.10 to -0.65) | 6487  (5133 to 8088) | 67.39  (53.14 to 83.25) | -0.59  (-0.69 to -0.48) |
| Guinea | 1765  (1343 to 2278) | 33.25  (25.19 to 43.30) | -0.01  (-0.01 to 0.00) | 173  (134 to 222) | 4.62  (3.53 to 5.92) | 0.73  (0.68 to 0.77) | 3687  (2870 to 4581) | 82.98  (65.41 to 102.29) | 0.40  (0.37 to 0.43) |
| Guinea-Bissau | 235  (179 to 299) | 34.48  (26.22 to 44.15) | 0.06  (0.05 to 0.08) | 16  (12 to 22) | 4.95  (3.73 to 6.54) | 0.62  (0.52 to 0.73) | 425  (329 to 533) | 87.98  (69.13 to 109.87) | 0.39  (0.33 to 0.45) |
| Guyana | 176  (134 to 230) | 29.99  (22.49 to 39.01) | -0.10  (-0.10 to -0.09) | 27  (21 to 36) | 6.54  (5.12 to 8.65) | 0.34  (0.08 to 0.60) | 558  (446 to 701) | 108.96  (88.07 to 136.65) | 0.20  (0.09 to 0.32) |
| Haiti | 1842  (1395 to 2378) | 28.98  (21.49 to 37.55) | 0.02  (0.01 to 0.02) | 237  (168 to 349) | 5.79  (4.19 to 8.31) | -0.43  (-0.50 to -0.36) | 5243  (3903 to 7242) | 98.37  (74.55 to 135.09) | -0.23  (-0.30 to -0.17) |
| Honduras | 1763  (1324 to 2266) | 30.57  (22.74 to 39.42) | 0.09  (0.06 to 0.11) | 227  (173 to 294) | 5.74  (4.27 to 7.56) | 1.62  (1.32 to 1.93) | 4523  (3679 to 5645) | 92.63  (74.86 to 116.14) | 1.19  (1.02 to 1.35) |
| Hungary | 12988  (9781 to 16920) | 73.54  (56.09 to 94.88) | -0.33  (-0.36 to -0.30) | 898  (717 to 1144) | 4.21  (3.36 to 5.37) | -0.44  (-0.50 to -0.39) | 26679  (20367 to 34890) | 131.80  (100.04 to 172.95) | -0.37  (-0.40 to -0.34) |
| Iceland | 321  (243 to 413) | 62.17  (47.16 to 79.59) | 0.27  (0.10 to 0.44) | 41  (32 to 52) | 5.78  (4.58 to 7.40) | -0.24  (-0.32 to -0.16) | 799  (638 to 1006) | 133.41  (105.29 to 168.57) | -0.10  (-0.20 to 0.00) |
| India | 702566  (532669 to 897841) | 62.29  (47.32 to 79.35) | 0.09  (0.08 to 0.10) | 29155  (22579 to 37300) | 3.99  (3.07 to 5.12) | 0.45  (0.32 to 0.59) | 1024772  (786262 to 1309013) | 105.66  (81.67 to 132.84) | 0.22  (0.17 to 0.27) |
| Indonesia | 142155  (107051 to 182356) | 66.74  (50.67 to 85.21) | 0.07  (0.06 to 0.07) | 4992  (3898 to 6556) | 4.33  (3.37 to 5.65) | 1.47  (1.40 to 1.53) | 192969  (143385 to 249120) | 113.21  (86.59 to 143.72) | 0.55  (0.53 to 0.57) |
| Iran  (Islamic Republic of) | 30247  (23219 to 38551) | 42.76  (32.42 to 55.06) | -0.14  (-0.29 to 0.01) | 1766  (1510 to 2003) | 3.16  (2.68 to 3.59) | 0.11  (-0.03 to 0.26) | 47997  (37710 to 60548) | 75.30  (59.93 to 94.58) | -0.06  (-0.23 to 0.10) |
| Iraq | 9518  (7286 to 12097) | 43.84  (33.03 to 56.02) | -0.03  (-0.06 to -0.01) | 630  (481 to 1022) | 4.43  (3.37 to 7.52) | 0.93  (0.78 to 1.08) | 18169  (14107 to 24797) | 96.62  (75.38 to 137.25) | 0.50  (0.43 to 0.57) |
| Ireland | 4524  (3419 to 5848) | 63.10  (48.00 to 80.65) | -0.53  (-0.62 to -0.44) | 483  (348 to 553) | 6.08  (4.38 to 6.95) | -0.39  (-0.55 to -0.22) | 10588  (8349 to 13245) | 137.38  (107.66 to 171.91) | -0.61  (-0.75 to -0.47) |
| Israel | 6217  (4664 to 8084) | 56.56  (42.84 to 72.52) | -0.34  (-0.38 to -0.31) | 642  (521 to 863) | 4.80  (3.91 to 6.44) | -1.17  (-1.32 to -1.02) | 14140  (11109 to 18197) | 116.32  (90.74 to 150.52) | -0.86  (-0.96 to -0.75) |
| Italy | 65057  (49548 to 83287) | 53.97  (41.05 to 69.02) | -0.85  (-0.93 to -0.76) | 10569  (8459 to 14449) | 5.23  (4.22 to 7.07) | -0.58  (-0.67 to -0.50) | 189787  (152217 to 238078) | 115.84  (91.37 to 147.56) | -0.78  (-0.84 to -0.71) |
| Jamaica | 913  (686 to 1174) | 30.50  (22.70 to 39.62) | 0.29  (0.26 to 0.32) | 197  (150 to 268) | 5.30  (4.03 to 7.38) | 0.51  (0.32 to 0.71) | 2991  (2386 to 3853) | 93.10  (74.03 to 120.71) | 0.51  (0.36 to 0.67) |
| Japan | 53883  (40560 to 69545) | 19.81  (15.36 to 25.34) | -2.01  (-2.44 to -1.58) | 13140  (9795 to 18259) | 2.39  (1.83 to 3.27) | -0.86  (-0.96 to -0.74) | 211572  (169138 to 263445) | 53.15  (42.54 to 66.74) | -1.14  (-1.33 to -0.96) |
| Jordan | 2567  (1963 to 3277) | 40.83  (30.84 to 52.28) | -0.03  (-0.04 to -0.02) | 139  (113 to 163) | 4.39  (3.44 to 5.19) | -0.18  (-0.36 to 0.00) | 4033  (3169 to 5063) | 85.19  (68.51 to 104.14) | -0.23  (-0.32 to -0.15) |
| Kazakhstan | 12262  (9261 to 15786) | 67.43  (51.31 to 86.52) | 0.10  (0.07 to 0.13) | 717  (559 to 1286) | 6.64  (5.18 to 11.75) | 1.06  (0.77 to 1.34) | 21272  (15655 to 29564) | 146.76  (110.39 to 207.20) | 0.48  (0.36 to 0.59) |
| Kenya | 4014  (3073 to 5161) | 19.39  (14.74 to 24.86) | 1.17  (0.56 to 1.77) | 600  (396 to 801) | 4.99  (3.23 to 6.74) | 1.44  (1.36 to 1.53) | 12426  (9173 to 15664) | 78.47  (56.47 to 99.88) | 1.28  (1.09 to 1.47) |
| Kiribati | 39  (29 to 51) | 60.42  (45.65 to 78.28) | 0.08  (0.05 to 0.12) | 2  (1 to 2) | 4.29  (3.36 to 5.47) | 0.12  (0.06 to 0.18) | 70  (54 to 88) | 124.71  (98.80 to 154.50) | -0.05  (-0.09 to -0.01) |
| Kuwait | 1078  (838 to 1352) | 41.51  (31.31 to 53.18) | 0.04  (0.03 to 0.05) | 38  (29 to 53) | 2.21  (1.67 to 3.04) | 0.33  (-0.02 to 0.68) | 1352  (1020 to 1792) | 64.70  (49.47 to 84.46) | 0.07  (-0.08 to 0.22) |
| Kyrgyzstan | 3017  (2302 to 3862) | 62.80  (48.12 to 79.41) | 0.04  (0.01 to 0.06) | 116  (89 to 134) | 3.67  (2.78 to 4.26) | 1.42  (0.75 to 2.10) | 4256  (3177 to 5591) | 108.95  (82.81 to 140.70) | 0.41  (0.20 to 0.62) |
| Lao People's Democratic Republic | 2548  (1939 to 3306) | 60.23  (45.33 to 78.19) | -0.04  (-0.05 to -0.03) | 90  (75 to 108) | 3.57  (2.98 to 4.23) | 0.96  (0.90 to 1.03) | 3504  (2643 to 4555) | 100.43  (77.33 to 127.89) | 0.37  (0.35 to 0.39) |
| Latvia | 2538  (1927 to 3243) | 72.02  (55.27 to 91.75) | 0.47  (0.35 to 0.59) | 197  (159 to 245) | 4.11  (3.32 to 5.12) | 0.45  (0.32 to 0.59) | 5436  (4163 to 6989) | 126.88  (95.98 to 164.14) | 0.48  (0.39 to 0.58) |
| Lebanon | 2210  (1652 to 2847) | 42.56  (31.75 to 54.97) | 0.07  (0.04 to 0.10) | 196  (124 to 255) | 4.07  (2.57 to 5.27) | -0.08  (-0.13 to -0.02) | 4428  (3336 to 5559) | 86.47  (65.25 to 108.85) | -0.07  (-0.08 to -0.05) |
| Lesotho | 410  (310 to 530) | 33.74  (25.51 to 43.27) | 0.06  (0.03 to 0.08) | 27  (20 to 35) | 3.94  (2.93 to 5.04) | 1.97  (1.76 to 2.17) | 774  (591 to 976) | 79.68  (61.39 to 99.60) | 1.10  (0.98 to 1.21) |
| Liberia | 709  (543 to 903) | 35.82  (27.20 to 45.94) | -0.10  (-0.13 to -0.07) | 51  (39 to 66) | 4.36  (3.32 to 5.64) | 0.59  (0.47 to 0.72) | 1210  (941 to 1528) | 80.71  (63.04 to 101.60) | 0.26  (0.18 to 0.34) |
| Libya | 2066  (1589 to 2605) | 42.37  (31.72 to 54.56) | 0.00  (-0.01 to 0.01) | 104  (69 to 142) | 2.66  (1.78 to 3.63) | 0.79  (0.68 to 0.91) | 3095  (2248 to 3981) | 70.76  (51.83 to 91.33) | 0.29  (0.26 to 0.33) |
| Lithuania | 3906  (2940 to 4993) | 76.29  (58.07 to 97.11) | 0.43  (0.39 to 0.47) | 291  (233 to 361) | 4.18  (3.36 to 5.19) | 0.24  (0.08 to 0.41) | 8224  (6208 to 10643) | 133.10  (99.48 to 174.78) | 0.35  (0.28 to 0.43) |
| Luxembourg | 665  (496 to 849) | 70.90  (53.21 to 90.73) | 0.09  (-0.15 to 0.34) | 69  (54 to 87) | 5.69  (4.45 to 7.19) | -0.28  (-0.40 to -0.17) | 1489  (1175 to 1889) | 140.20  (109.56 to 179.75) | -0.16  (-0.32 to 0.00) |
| Madagascar | 1896  (1431 to 2447) | 19.01  (14.32 to 24.64) | 0.02  (0.01 to 0.04) | 323  (206 to 429) | 5.67  (3.62 to 7.54) | -0.07  (-0.16 to 0.03) | 7346  (5264 to 9513) | 93.69  (65.65 to 120.29) | -0.03  (-0.12 to 0.06) |
| Malawi | 1371  (1050 to 1771) | 20.01  (15.17 to 25.80) | 0.02  (0.01 to 0.02) | 185  (111 to 242) | 4.18  (2.48 to 5.54) | 0.62  (0.54 to 0.69) | 3897  (2642 to 4943) | 70.07  (46.97 to 87.90) | 0.38  (0.34 to 0.43) |
| Malaysia | 16610  (12646 to 21337) | 62.34  (47.56 to 79.36) | 0.05  (-0.01 to 0.11) | 721  (561 to 932) | 4.05  (3.12 to 5.31) | 0.89  (0.55 to 1.23) | 25181  (19097 to 32307) | 109.70  (84.50 to 139.98) | 0.38  (0.26 to 0.50) |
| Maldives | 178  (138 to 226) | 59.08  (44.88 to 75.90) | 0.03  (0.02 to 0.04) | 8  (6 to 10) | 3.89  (2.94 to 4.72) | 0.05  (-0.05 to 0.15) | 250  (188 to 327) | 99.21  (75.87 to 127.04) | -0.05  (-0.09 to -0.01) |
| Mali | 2776  (2123 to 3580) | 33.67  (25.57 to 43.44) | 0.03  (0.02 to 0.04) | 234  (172 to 292) | 4.87  (3.56 to 6.07) | 0.49  (0.40 to 0.59) | 5353  (4173 to 6727) | 84.82  (66.20 to 105.27) | 0.32  (0.27 to 0.37) |
| Malta | 516  (383 to 674) | 60.81  (46.15 to 78.24) | -0.11  (-0.18 to -0.04) | 45  (36 to 55) | 4.24  (3.38 to 5.21) | -0.40  (-0.47 to -0.34) | 1131  (887 to 1444) | 114.90  (89.45 to 147.87) | -0.33  (-0.37 to -0.29) |
| Marshall Islands | 19  (14 to 25) | 58.74  (44.47 to 75.36) | 0.09  (0.07 to 0.10) | 1  (1 to 1) | 6.15  (4.61 to 7.99) | 0.32  (0.21 to 0.42) | 40  (30 to 51) | 147.09  (114.96 to 187.12) | 0.28  (0.21 to 0.35) |
| Mauritania | 713  (548 to 909) | 35.08  (26.70 to 44.79) | 0.05  (0.03 to 0.07) | 64  (48 to 80) | 4.61  (3.47 to 5.71) | 0.02  (-0.02 to 0.07) | 1410  (1110 to 1757) | 83.45  (66.01 to 103.11) | 0.00  (-0.03 to 0.03) |
| Mauritius | 1079  (813 to 1395) | 60.99  (46.24 to 77.84) | 0.00  (-0.01 to 0.01) | 54  (43 to 71) | 3.65  (2.88 to 4.76) | 0.21  (0.09 to 0.34) | 1703  (1312 to 2192) | 103.71  (80.14 to 133.00) | 0.01  (-0.06 to 0.09) |
| Mexico | 37281  (28242 to 47903) | 32.61  (24.42 to 42.11) | 0.02  (0.00 to 0.03) | 5228  (4191 to 7002) | 5.23  (4.17 to 7.05) | -0.19  (-0.34 to -0.04) | 99070  (81768 to 122854) | 91.86  (75.82 to 114.22) | -0.03  (-0.11 to 0.04) |
| Micronesia  (Federated States of) | 38  (28 to 50) | 57.05  (42.95 to 73.12) | -0.03  (-0.04 to -0.01) | 3  (2 to 4) | 7.08  (5.45 to 9.35) | 0.59  (0.58 to 0.61) | 86  (65 to 110) | 156.79  (122.38 to 198.07) | 0.37  (0.36 to 0.38) |
| Monaco | 49  (37 to 64) | 60.34  (45.58 to 77.29) | -0.54  (-0.59 to -0.49) | 7  (5 to 8) | 4.96  (3.87 to 5.96) | 0.13  (0.04 to 0.22) | 130  (102 to 162) | 122.34  (95.13 to 155.06) | -0.27  (-0.29 to -0.26) |
| Mongolia | 1650  (1244 to 2144) | 65.97  (50.24 to 84.40) | 0.13  (0.11 to 0.14) | 51  (41 to 63) | 4.99  (4.11 to 5.94) | 0.15  (-0.04 to 0.34) | 2208  (1649 to 2886) | 127.39  (98.98 to 161.65) | 0.01  (-0.09 to 0.12) |
| Montenegro | 671  (508 to 869) | 68.05  (51.70 to 87.00) | -0.23  (-0.27 to -0.20) | 121  (97 to 160) | 14.38  (11.31 to 19.38) | 1.13  (0.96 to 1.30) | 2377  (1955 to 2959) | 250.62  (205.82 to 313.72) | 0.62  (0.53 to 0.71) |
| Morocco | 12757  (9680 to 16337) | 42.50  (31.99 to 54.68) | -0.07  (-0.08 to -0.06) | 874  (665 to 1082) | 4.37  (3.20 to 5.55) | 1.25  (1.06 to 1.44) | 22500  (17710 to 28189) | 89.32  (70.27 to 110.18) | 0.56  (0.48 to 0.64) |
| Mozambique | 2024  (1538 to 2610) | 19.86  (14.97 to 25.64) | 0.00  (-0.01 to 0.02) | 326  (192 to 478) | 5.33  (3.08 to 7.76) | 1.34  (1.24 to 1.44) | 6734  (4412 to 9338) | 84.39  (54.17 to 116.32) | 1.03  (0.94 to 1.11) |
| Myanmar | 27210  (20581 to 34856) | 59.78  (45.33 to 75.89) | -0.03  (-0.04 to -0.02) | 1114  (911 to 1384) | 3.56  (2.88 to 4.40) | 0.94  (0.88 to 1.01) | 39276  (29923 to 50422) | 99.70  (76.99 to 126.11) | 0.32  (0.29 to 0.35) |
| Namibia | 461  (354 to 589) | 33.71  (25.41 to 43.02) | 0.13  (0.08 to 0.17) | 40  (30 to 51) | 4.10  (3.08 to 5.16) | 0.61  (0.44 to 0.78) | 959  (746 to 1200) | 80.95  (63.56 to 100.37) | 0.33  (0.23 to 0.43) |
| Nauru | 2  (2 to 3) | 60.49  (45.66 to 78.40) | 0.06  (0.03 to 0.09) | 0  (0 to 0) | 6.25  (4.90 to 7.96) | 0.13  (-0.04 to 0.30) | 5  (3 to 6) | 150.32  (117.66 to 190.06) | 0.08  (-0.05 to 0.21) |
| Nepal | 12082  (9146 to 15356) | 54.67  (41.51 to 69.51) | -0.10  (-0.13 to -0.07) | 569  (377 to 757) | 4.08  (2.67 to 5.52) | 1.99  (1.88 to 2.10) | 18597  (14007 to 24134) | 100.29  (75.42 to 129.72) | 0.71  (0.66 to 0.75) |
| Netherlands | 17570  (13107 to 22884) | 55.97  (42.51 to 71.94) | -0.28  (-0.34 to -0.22) | 2065  (1673 to 2677) | 5.27  (4.27 to 6.83) | -0.63  (-0.74 to -0.52) | 43581  (34852 to 55605) | 120.30  (95.51 to 153.90) | -0.43  (-0.53 to -0.33) |
| New Zealand | 6855  (5352 to 8598) | 91.27  (72.31 to 113.39) | -0.19  (-0.29 to -0.08) | 659  (519 to 794) | 7.21  (5.71 to 8.71) | -0.08  (-0.22 to 0.06) | 14563  (11633 to 18207) | 175.07  (139.28 to 218.70) | -0.20  (-0.23 to -0.17) |
| Nicaragua | 1284  (975 to 1670) | 30.50  (22.78 to 39.69) | 0.08  (0.06 to 0.10) | 190  (160 to 225) | 6.52  (5.45 to 7.64) | 1.40  (0.79 to 2.01) | 3508  (2940 to 4158) | 99.89  (84.02 to 117.79) | 0.84  (0.47 to 1.23) |
| Niger | 2484  (1881 to 3194) | 33.48  (25.20 to 43.05) | -0.06  (-0.07 to -0.05) | 145  (98 to 195) | 4.07  (2.70 to 5.48) | 0.52  (0.45 to 0.59) | 4125  (3042 to 5355) | 77.14  (56.98 to 98.57) | 0.27  (0.23 to 0.31) |
| Nigeria | 31056  (23849 to 39780) | 37.13  (28.17 to 47.52) | 0.10  (0.03 to 0.17) | 2367  (1779 to 2904) | 4.81  (3.58 to 5.91) | -0.42  (-0.52 to -0.32) | 55781  (43338 to 69220) | 87.69  (68.73 to 107.82) | -0.21  (-0.27 to -0.15) |
| Niue | 1  (1 to 2) | 61.05  (46.41 to 78.30) | 0.11  (0.08 to 0.13) | 0  (0 to 0) | 5.05  (3.75 to 6.36) | -0.05  (-0.08 to -0.01) | 3  (2 to 4) | 130.40  (103.33 to 164.13) | 0.02  (-0.01 to 0.06) |
| North Macedonia | 2101  (1568 to 2713) | 63.12  (47.72 to 80.61) | -0.13  (-0.14 to -0.12) | 162  (133 to 196) | 8.22  (6.78 to 9.93) | 0.23  (0.12 to 0.33) | 4438  (3517 to 5601) | 159.84  (129.27 to 197.35) | 0.00  (-0.03 to 0.03) |
| Northern Mariana Islands | 32  (24 to 42) | 60.57  (45.86 to 77.16) | 0.08  (0.05 to 0.12) | 2  (2 to 2) | 6.73  (5.63 to 7.83) | 0.57  (0.27 to 0.88) | 64  (51 to 78) | 153.03  (125.88 to 182.92) | 0.37  (0.19 to 0.55) |
| Norway | 5177  (3923 to 6668) | 59.38  (45.29 to 75.42) | -0.44  (-0.48 to -0.40) | 824  (542 to 936) | 6.76  (4.49 to 7.69) | 0.18  (0.05 to 0.31) | 14446  (11281 to 17668) | 138.06  (108.02 to 170.33) | -0.30  (-0.37 to -0.23) |
| Oman | 745  (576 to 944) | 42.61  (32.31 to 54.88) | 0.30  (0.26 to 0.34) | 41  (32 to 49) | 8.38  (5.14 to 10.31) | 0.43  (0.19 to 0.67) | 1094  (871 to 1367) | 120.67  (95.12 to 145.85) | 0.29  (0.16 to 0.42) |
| Pakistan | 65459  (49361 to 83662) | 61.52  (46.48 to 78.21) | 0.02  (0.01 to 0.03) | 2713  (2080 to 3440) | 4.50  (3.41 to 5.79) | 0.96  (0.80 to 1.12) | 95095  (72530 to 121183) | 112.61  (87.07 to 141.19) | 0.39  (0.32 to 0.47) |
| Palau | 13  (10 to 17) | 61.29  (46.51 to 78.41) | 0.10  (0.08 to 0.13) | 0  (0 to 0) | 3.20  (2.53 to 3.87) | -0.19  (-0.29 to -0.09) | 18  (14 to 24) | 104.56  (81.28 to 132.36) | -0.04  (-0.07 to -0.01) |
| Palestine | 894  (685 to 1144) | 40.09  (30.41 to 51.31) | 0.02  (0.01 to 0.02) | 64  (52 to 89) | 4.64  (3.74 to 6.60) | 0.53  (0.39 to 0.68) | 1685  (1343 to 2147) | 92.92  (74.60 to 117.47) | 0.26  (0.19 to 0.34) |
| Panama | 1272  (956 to 1638) | 30.77  (22.97 to 39.80) | 0.18  (0.15 to 0.20) | 180  (131 to 241) | 3.99  (2.93 to 5.39) | 0.31  (0.20 to 0.42) | 3109  (2443 to 3933) | 73.50  (57.60 to 93.12) | 0.22  (0.17 to 0.27) |
| Papua New Guinea | 2590  (1946 to 3325) | 58.06  (43.98 to 73.68) | 0.06  (0.04 to 0.08) | 102  (67 to 151) | 4.01  (2.66 to 5.84) | 0.34  (0.32 to 0.37) | 4112  (3014 to 5473) | 111.18  (83.70 to 145.80) | 0.33  (0.31 to 0.35) |
| Paraguay | 2179  (1648 to 2813) | 40.08  (30.31 to 51.88) | -0.05  (-0.07 to -0.03) | 240  (180 to 311) | 4.69  (3.54 to 6.09) | 0.30  (0.18 to 0.42) | 4959  (3936 to 6207) | 95.45  (75.89 to 119.73) | 0.30  (0.23 to 0.37) |
| Peru | 4748  (3536 to 6147) | 14.92  (11.14 to 19.42) | 0.20  (0.16 to 0.24) | 1177  (882 to 1538) | 3.52  (2.65 to 4.62) | -0.83  (-1.01 to -0.63) | 17548  (13728 to 21939) | 54.39  (42.48 to 68.20) | -0.58  (-0.70 to -0.45) |
| Philippines | 46683  (35439 to 60065) | 61.56  (46.65 to 78.41) | -0.02  (-0.04 to -0.01) | 1571  (1299 to 2070) | 3.25  (2.68 to 4.25) | 1.28  (1.04 to 1.52) | 62919  (47210 to 82568) | 97.53  (74.38 to 126.25) | 0.59  (0.48 to 0.70) |
| Poland | 49678  (37690 to 64047) | 74.60  (57.04 to 95.05) | 0.27  (0.02 to 0.53) | 4090  (3114 to 4975) | 5.33  (4.05 to 6.47) | 0.39  (0.32 to 0.47) | 106704  (83133 to 135429) | 146.05  (113.54 to 186.37) | 0.34  (0.15 to 0.53) |
| Portugal | 12564  (9532 to 16238) | 60.86  (46.41 to 78.71) | -0.71  (-0.75 to -0.65) | 1253  (1013 to 1861) | 3.97  (3.23 to 5.84) | -1.05  (-1.17 to -0.94) | 29239  (22636 to 38168) | 110.87  (84.50 to 145.72) | -0.84  (-0.91 to -0.77) |
| Puerto Rico | 2225  (1620 to 2907) | 30.81  (23.01 to 39.89) | 0.09  (0.08 to 0.11) | 370  (264 to 469) | 3.88  (2.81 to 4.93) | -0.25  (-0.33 to -0.17) | 5941  (4678 to 7426) | 73.03  (57.64 to 92.08) | -0.12  (-0.18 to -0.07) |
| Qatar | 455  (341 to 588) | 40.83  (31.10 to 52.17) | -0.12  (-0.13 to -0.10) | 21  (15 to 28) | 12.12  (9.13 to 16.76) | 0.06  (-0.27 to 0.38) | 753  (565 to 975) | 178.86  (142.32 to 228.96) | -0.28  (-0.47 to -0.09) |
| Republic of Korea | 21840  (16460 to 28433) | 24.56  (18.62 to 31.52) | -0.45  (-0.50 to -0.40) | 2022  (1640 to 2302) | 2.57  (2.05 to 2.94) | -0.45  (-0.50 to -0.39) | 49455  (39535 to 61932) | 56.82  (45.46 to 70.80) | -0.53  (-0.58 to -0.49) |
| Republic of Moldova | 4200  (3213 to 5413) | 72.59  (55.75 to 92.75) | 0.04  (-0.02 to 0.11) | 196  (156 to 247) | 3.41  (2.72 to 4.29) | -0.83  (-1.13 to -0.53) | 7005  (5189 to 9336) | 120.03  (89.33 to 159.45) | -0.20  (-0.30 to -0.10) |
| Romania | 22980  (17422 to 29484) | 66.84  (51.05 to 85.75) | -0.65  (-0.81 to -0.48) | 1473  (933 to 1773) | 3.61  (2.31 to 4.34) | -0.13  (-0.21 to -0.04) | 45680  (34325 to 59763) | 116.90  (87.37 to 153.71) | -0.45  (-0.58 to -0.32) |
| Russian Federation | 172376  (130633 to 221093) | 73.58  (56.52 to 94.37) | 0.36  (0.33 to 0.39) | 9962  (8166 to 13099) | 4.27  (3.50 to 5.65) | 0.23  (0.03 to 0.42) | 312583  (237566 to 408735) | 130.90  (99.50 to 170.90) | 0.37  (0.28 to 0.46) |
| Rwanda | 1065  (815 to 1361) | 18.88  (14.32 to 24.37) | -0.12  (-0.13 to -0.11) | 168  (105 to 219) | 5.02  (3.08 to 6.60) | -0.27  (-0.49 to -0.04) | 3486  (2448 to 4373) | 79.35  (54.68 to 99.08) | -0.55  (-0.75 to -0.34) |
| Saint Kitts and Nevis | 19  (14 to 25) | 30.29  (22.64 to 39.61) | 0.12  (0.11 to 0.13) | 4  (4 to 5) | 10.06  (8.48 to 12.53) | 0.23  (0.06 to 0.41) | 75  (63 to 94) | 146.73  (125.35 to 182.39) | -0.07  (-0.21 to 0.06) |
| Saint Lucia | 65  (48 to 83) | 30.40  (22.77 to 39.28) | 0.01  (0.00 to 0.02) | 14  (12 to 18) | 7.55  (6.10 to 9.43) | -0.79  (-1.09 to -0.48) | 222  (185 to 270) | 110.01  (91.89 to 133.88) | -0.62  (-0.84 to -0.39) |
| Saint Vincent and the Grenadines | 41  (30 to 53) | 30.51  (22.80 to 39.50) | 0.23  (0.22 to 0.24) | 8  (6 to 10) | 7.02  (5.84 to 9.12) | 0.49  (0.16 to 0.83) | 132  (111 to 164) | 108.12  (91.09 to 133.85) | 0.30  (0.06 to 0.54) |
| Samoa | 88  (67 to 113) | 62.27  (46.94 to 79.98) | 0.07  (0.05 to 0.09) | 6  (4 to 7) | 4.94  (3.87 to 6.22) | -0.18  (-0.32 to -0.04) | 170  (133 to 213) | 129.58  (101.89 to 162.28) | -0.03  (-0.08 to 0.03) |
| San Marino | 33  (25 to 43) | 59.74  (45.01 to 76.68) | -0.56  (-0.59 to -0.52) | 5  (3 to 6) | 5.24  (3.71 to 6.99) | 0.38  (0.27 to 0.50) | 89  (67 to 115) | 123.64  (91.42 to 160.96) | -0.12  (-0.16 to -0.08) |
| Sao Tome and Principe | 38  (29 to 49) | 37.36  (28.27 to 48.03) | 0.07  (0.01 to 0.12) | 3  (2 to 4) | 5.03  (3.83 to 6.10) | 1.29  (1.16 to 1.43) | 72  (58 to 90) | 90.16  (72.25 to 111.18) | 0.76  (0.72 to 0.80) |
| Saudi Arabia | 6856  (5264 to 8687) | 39.79  (30.10 to 50.76) | 0.03  (0.00 to 0.05) | 301  (236 to 384) | 4.29  (3.35 to 5.29) | -0.04  (-0.21 to 0.14) | 9816  (7548 to 12763) | 85.11  (66.98 to 105.14) | -0.02  (-0.11 to 0.08) |
| Senegal | 2542  (1935 to 3280) | 34.94  (26.49 to 44.97) | -0.06  (-0.07 to -0.05) | 210  (156 to 267) | 4.55  (3.35 to 5.80) | 0.71  (0.66 to 0.77) | 4825  (3767 to 6079) | 82.46  (64.54 to 103.12) | 0.38  (0.34 to 0.42) |
| Serbia | 9539  (7131 to 12451) | 62.65  (47.59 to 80.41) | -0.29  (-0.43 to -0.15) | 803  (650 to 1111) | 6.28  (5.08 to 8.77) | 0.63  (0.49 to 0.78) | 21640  (16702 to 28024) | 139.63  (108.59 to 179.45) | 0.05  (-0.04 to 0.13) |
| Seychelles | 67  (51 to 86) | 61.61  (46.92 to 78.48) | -0.07  (-0.08 to -0.05) | 3  (2 to 5) | 4.17  (2.87 to 5.56) | 0.35  (0.27 to 0.43) | 106  (80 to 137) | 108.85  (82.20 to 139.96) | 0.06  (0.02 to 0.10) |
| Sierra Leone | 1304  (989 to 1669) | 37.62  (28.33 to 48.49) | -0.13  (-0.16 to -0.09) | 84  (65 to 111) | 3.98  (3.07 to 5.29) | 0.72  (0.65 to 0.81) | 2194  (1671 to 2771) | 79.39  (61.49 to 100.19) | 0.32  (0.28 to 0.36) |
| Singapore | 2084  (1597 to 2685) | 25.58  (19.54 to 32.81) | -0.28  (-0.31 to -0.24) | 135  (99 to 163) | 1.97  (1.44 to 2.37) | -1.08  (-1.25 to -0.91) | 3935  (3015 to 5113) | 51.94  (40.06 to 67.04) | -0.78  (-0.84 to -0.72) |
| Slovakia | 6844  (5180 to 8824) | 75.46  (57.57 to 96.25) | 0.19  (-0.06 to 0.44) | 457  (366 to 557) | 5.17  (4.13 to 6.30) | 0.22  (0.12 to 0.32) | 13586  (10485 to 17529) | 146.25  (112.91 to 187.67) | 0.10  (-0.09 to 0.30) |
| Slovenia | 2555  (1935 to 3289) | 66.05  (50.50 to 85.08) | -0.22  (-0.24 to -0.19) | 236  (165 to 304) | 4.37  (3.04 to 5.66) | -0.19  (-0.34 to -0.04) | 5800  (4422 to 7500) | 124.96  (94.67 to 162.20) | -0.25  (-0.33 to -0.17) |
| Solomon Islands | 165  (124 to 211) | 57.90  (43.67 to 73.72) | 0.03  (0.02 to 0.04) | 8  (6 to 11) | 4.59  (3.47 to 6.03) | 0.79  (0.74 to 0.86) | 315  (237 to 425) | 127.94  (98.35 to 163.44) | 0.38  (0.34 to 0.42) |
| Somalia | 1113  (838 to 1454) | 18.56  (14.06 to 24.18) | 0.04  (0.03 to 0.05) | 110  (53 to 163) | 3.70  (1.69 to 5.50) | 0.03  (-0.11 to 0.16) | 2878  (1777 to 3966) | 64.46  (37.68 to 89.04) | -0.15  (-0.25 to -0.05) |
| South Africa | 16649  (12712 to 21269) | 37.98  (28.89 to 48.71) | -0.06  (-0.07 to -0.04) | 1206  (1039 to 1350) | 3.96  (3.37 to 4.42) | 0.80  (0.57 to 1.04) | 30445  (24786 to 37352) | 80.28  (66.26 to 97.20) | 0.33  (0.20 to 0.47) |
| South Sudan | 695  (531 to 894) | 19.34  (14.59 to 25.02) | 0.11  (0.09 to 0.14) | 86  (50 to 125) | 3.75  (2.18 to 5.39) | -0.49  (-0.62 to -0.36) | 1883  (1285 to 2528) | 64.93  (43.60 to 87.68) | -0.45  (-0.56 to -0.33) |
| Spain | 49538  (37960 to 62814) | 59.99  (45.96 to 76.33) | -0.64  (-0.81 to -0.46) | 7378  (5887 to 9278) | 5.41  (4.39 to 6.83) | -0.46  (-0.53 to -0.39) | 135054  (108181 to 168601) | 123.88  (97.64 to 157.71) | -0.60  (-0.72 to -0.47) |
| Sri Lanka | 15361  (11530 to 19942) | 59.37  (45.01 to 75.77) | 0.07  (0.06 to 0.08) | 599  (454 to 758) | 3.40  (2.53 to 4.30) | 1.31  (1.07 to 1.55) | 21409  (16192 to 28046) | 94.03  (71.76 to 122.08) | 0.46  (0.38 to 0.54) |
| Sudan | 7376  (5632 to 9398) | 41.83  (31.36 to 53.78) | 0.12  (0.12 to 0.13) | 400  (299 to 516) | 3.19  (2.35 to 4.14) | 0.61  (0.55 to 0.68) | 11806  (8970 to 15215) | 77.82  (59.82 to 99.82) | 0.32  (0.28 to 0.35) |
| Suriname | 178  (133 to 228) | 30.38  (22.66 to 39.25) | 0.15  (0.13 to 0.17) | 26  (21 to 32) | 5.08  (4.06 to 6.16) | 0.14  (-0.15 to 0.42) | 495  (408 to 597) | 89.87  (73.93 to 107.84) | 0.18  (0.02 to 0.34) |
| Sweden | 15505  (11645 to 20101) | 88.44  (67.69 to 112.96) | 0.29  (0.00 to 0.58) | 2451  (1110 to 2943) | 8.63  (4.00 to 10.32) | 1.46  (1.33 to 1.60) | 44218  (29996 to 56073) | 188.07  (132.83 to 241.02) | 0.66  (0.48 to 0.86) |
| Switzerland | 7830  (5914 to 10029) | 50.94  (38.68 to 65.54) | -0.15  (-0.45 to 0.16) | 796  (618 to 954) | 3.38  (2.66 to 4.05) | -0.22  (-0.31 to -0.12) | 17616  (13769 to 22507) | 92.69  (71.50 to 120.19) | -0.18  (-0.38 to 0.03) |
| Syrian Arab Republic | 4919  (3676 to 6345) | 41.11  (30.95 to 52.75) | -0.03  (-0.04 to -0.02) | 297  (218 to 385) | 4.63  (3.35 to 5.95) | 0.58  (0.49 to 0.66) | 8477  (6460 to 10816) | 90.57  (69.49 to 113.06) | 0.20  (0.15 to 0.26) |
| Taiwan  (Province of China) | 20830  (16232 to 25933) | 52.39  (41.15 to 64.85) | -0.21  (-0.23 to -0.18) | 1399  (1070 to 1946) | 3.32  (2.54 to 4.61) | 0.40  (0.32 to 0.49) | 35193  (26881 to 45210) | 87.09  (66.54 to 111.88) | -0.07  (-0.12 to -0.03) |
| Tajikistan | 3406  (2572 to 4430) | 61.77  (47.30 to 78.34) | 0.02  (0.00 to 0.03) | 143  (112 to 172) | 7.33  (5.59 to 8.78) | 0.96  (0.45 to 1.48) | 4837  (3696 to 6222) | 147.07  (117.62 to 182.69) | 0.69  (0.53 to 0.86) |
| Thailand | 60180  (45197 to 77100) | 58.36  (44.07 to 74.50) | 0.07  (0.07 to 0.08) | 3483  (2558 to 4434) | 3.63  (2.66 to 4.62) | -0.72  (-0.83 to -0.60) | 94884  (71374 to 123673) | 95.56  (72.27 to 124.94) | -0.24  (-0.29 to -0.19) |
| Timor-Leste | 473  (355 to 617) | 58.64  (44.42 to 74.85) | -0.02  (-0.04 to -0.01) | 16  (13 to 21) | 3.49  (2.73 to 4.43) | 1.93  (1.81 to 2.06) | 654  (485 to 869) | 96.73  (72.81 to 126.94) | 0.73  (0.67 to 0.81) |
| Togo | 1214  (913 to 1562) | 34.45  (25.83 to 44.45) | -0.08  (-0.09 to -0.07) | 81  (63 to 105) | 4.89  (3.72 to 6.32) | 0.56  (0.53 to 0.58) | 2125  (1640 to 2657) | 86.61  (68.31 to 106.93) | 0.28  (0.27 to 0.29) |
| Tokelau | 1  (1 to 1) | 60.94  (46.48 to 78.56) | 0.12  (0.10 to 0.14) | 0  (0 to 0) | 4.87  (3.86 to 6.00) | 0.33  (0.27 to 0.38) | 2  (1 to 2) | 127.86  (101.81 to 159.67) | 0.16  (0.13 to 0.19) |
| Tonga | 49  (37 to 62) | 62.94  (47.20 to 80.39) | 0.13  (0.10 to 0.16) | 3  (2 to 4) | 4.14  (3.09 to 5.28) | 0.31  (0.17 to 0.44) | 90  (70 to 115) | 117.17  (91.79 to 150.11) | 0.24  (0.17 to 0.31) |
| Trinidad and Tobago | 579  (432 to 756) | 31.19  (23.24 to 40.17) | 0.08  (0.07 to 0.09) | 75  (53 to 97) | 4.46  (3.16 to 5.73) | 0.36  (0.21 to 0.52) | 1461  (1115 to 1833) | 82.78  (63.32 to 104.00) | 0.27  (0.19 to 0.36) |
| Tunisia | 5031  (3800 to 6504) | 40.51  (30.45 to 52.06) | 0.05  (0.04 to 0.06) | 383  (280 to 502) | 3.90  (2.83 to 5.07) | 0.61  (0.50 to 0.72) | 9335  (7127 to 11851) | 82.77  (63.74 to 104.79) | 0.32  (0.27 to 0.38) |
| Turkey | 37208  (27994 to 47645) | 42.63  (32.10 to 54.55) | -0.37  (-0.46 to -0.27) | 2416  (1886 to 3073) | 3.15  (2.43 to 3.97) | -0.65  (-0.89 to -0.41) | 61729  (47405 to 79021) | 74.82  (57.84 to 95.30) | -0.70  (-0.78 to -0.61) |
| Turkmenistan | 2697  (2034 to 3462) | 65.43  (49.85 to 83.60) | 0.16  (0.14 to 0.18) | 126  (100 to 171) | 4.65  (3.70 to 6.27) | 0.87  (0.68 to 1.07) | 4315  (3289 to 5581) | 130.65  (100.58 to 167.74) | 0.56  (0.46 to 0.66) |
| Tuvalu | 6  (5 to 8) | 60.55  (45.76 to 77.78) | 0.10  (0.08 to 0.13) | 0  (0 to 0) | 5.33  (4.22 to 6.75) | -0.18  (-0.26 to -0.10) | 12  (10 to 15) | 135.04  (106.88 to 169.56) | -0.07  (-0.10 to -0.03) |
| Uganda | 2504  (1913 to 3213) | 18.97  (14.43 to 24.53) | -0.12  (-0.14 to -0.11) | 387  (213 to 536) | 4.63  (2.49 to 6.47) | 0.82  (0.80 to 0.85) | 7937  (4967 to 10423) | 74.91  (46.54 to 98.31) | 0.49  (0.47 to 0.52) |
| Ukraine | 57171  (43611 to 73280) | 76.81  (59.30 to 98.35) | 0.37  (0.32 to 0.41) | 4435  (3632 to 5949) | 5.90  (4.79 to 7.92) | 0.24  (0.10 to 0.38) | 121591  (94110 to 156580) | 155.92  (121.12 to 200.19) | 0.38  (0.30 to 0.46) |
| United Arab Emirates | 1843  (1368 to 2395) | 41.75  (31.45 to 53.07) | -0.19  (-0.22 to -0.16) | 38  (21 to 72) | 4.12  (2.25 to 7.16) | -0.29  (-0.91 to 0.33) | 2214  (1470 to 3300) | 90.02  (60.43 to 129.24) | -0.26  (-0.63 to 0.10) |
| United Kingdom | 83536  (64908 to 104380) | 75.05  (59.02 to 93.07) | -0.03  (-0.13 to 0.07) | 9031  (6630 to 10127) | 5.86  (4.33 to 6.56) | 0.16  (0.07 to 0.24) | 199715  (158674 to 248134) | 148.14  (116.72 to 184.99) | 0.08  (0.01 to 0.14) |
| United Republic of Tanzania | 5201  (4013 to 6719) | 22.26  (16.88 to 29.00) | -0.06  (-0.18 to 0.06) | 820  (521 to 1064) | 5.11  (3.21 to 6.70) | 0.05  (-0.04 to 0.15) | 16457  (11439 to 20869) | 84.74  (58.40 to 106.72) | -0.06  (-0.16 to 0.04) |
| United States of America | 607599  (489249 to 736141) | 109.52  (88.97 to 131.92) | 1.51  (1.05 to 1.97) | 33211  (26673 to 40280) | 5.08  (4.12 to 6.16) | 0.85  (0.77 to 0.94) | 955312  (754899 to 1198324) | 162.34  (127.34 to 203.97) | 1.02  (0.81 to 1.23) |
| United States Virgin Islands | 57  (41 to 75) | 29.94  (22.46 to 38.63) | 0.02  (0.00 to 0.03) | 11  (9 to 13) | 7.46  (6.24 to 8.97) | 1.22  (1.03 to 1.41) | 196  (166 to 231) | 115.41  (98.60 to 135.98) | 0.87  (0.73 to 1.03) |
| Uruguay | 2208  (1649 to 2880) | 42.56  (32.27 to 55.30) | 0.02  (0.00 to 0.04) | 322  (216 to 368) | 4.62  (3.13 to 5.27) | 0.15  (0.03 to 0.28) | 5733  (4515 to 7038) | 93.87  (73.79 to 116.24) | 0.03  (-0.03 to 0.08) |
| Uzbekistan | 14472  (10861 to 18790) | 62.18  (47.57 to 78.93) | 0.23  (0.21 to 0.25) | 504  (419 to 628) | 8.60  (6.78 to 10.05) | 4.58  (4.21 to 4.96) | 20563  (15839 to 26570) | 164.01  (134.73 to 200.80) | 2.32  (2.16 to 2.48) |
| Vanuatu | 108  (81 to 140) | 64.46  (48.86 to 82.64) | 0.00  (-0.01 to 0.01) | 5  (4 to 6) | 4.32  (3.20 to 5.74) | 0.73  (0.68 to 0.79) | 186  (141 to 245) | 125.63  (95.86 to 161.32) | 0.38  (0.34 to 0.42) |
| Venezuela  (Bolivarian Republic of) | 9179  (6879 to 11891) | 32.04  (24.04 to 41.70) | -0.09  (-0.12 to -0.06) | 1169  (862 to 1511) | 4.39  (3.26 to 5.69) | 0.04  (-0.12 to 0.20) | 21986  (17333 to 27579) | 81.03  (64.06 to 101.17) | 0.02  (-0.07 to 0.11) |
| Viet Nam | 58075  (44556 to 73275) | 63.30  (48.03 to 80.39) | 0.44  (0.39 to 0.49) | 3124  (2391 to 4042) | 4.63  (3.53 to 5.96) | 0.67  (0.56 to 0.78) | 91404  (69115 to 116525) | 114.08  (86.71 to 144.54) | 0.53  (0.48 to 0.59) |
| Yemen | 4946  (3813 to 6328) | 39.69  (30.03 to 51.24) | -0.02  (-0.04 to 0.00) | 255  (201 to 334) | 3.34  (2.63 to 4.32) | 0.66  (0.59 to 0.73) | 7951  (5992 to 10322) | 77.67  (59.98 to 99.56) | 0.29  (0.26 to 0.33) |
| Zambia | 1170  (893 to 1506) | 18.80  (14.21 to 24.21) | -0.08  (-0.10 to -0.07) | 242  (166 to 308) | 6.43  (4.49 to 8.10) | 1.56  (1.33 to 1.80) | 4873  (3513 to 6011) | 99.95  (72.15 to 122.67) | 1.25  (1.07 to 1.43) |
| Zimbabwe | 2202  (1668 to 2836) | 33.09  (24.99 to 42.30) | -0.02  (-0.04 to 0.01) | 129  (73 to 165) | 3.80  (1.94 to 4.86) | 0.50  (0.36 to 0.64) | 3555  (2690 to 4478) | 71.95  (54.32 to 89.59) | 0.25  (0.16 to 0.33) |

ASIR, age-standardized incident rates; ASDR, age-standardized death rates; DALY, disability adjusted life-year; UI, uncertain interval; EAPC, estimated annual percentage change; CI, confidence interval.
