# Supplementary material for: Burden of atrial fibrillation and its attributable risk factors from 1990 to 2019: An analysis of the Global Burden of Disease study 2019
Source: Front Cardiovasc Med. 2022 Oct 26;9:997698. doi: 10.3389/fcvm.2022.997698 (PMC9643162; doi:10.3389/fcvm.2022.997698)
Supplement: Supplementary file 2 [file Table_2.docx]

Figure legend

Supplementary Figure 1. The number of incident cases (A), death cases (B), and disability-adjusted life-years (DALYs) (C) of atrial fibrillation in 204 countries and territories in 2019.

Supplementary Figure 2. The age-standardized rates of atrial fibrillation in 204 countries and territories in 2019. (A) Age-standardized incidence rate. (B) Age-standardized death rate (ASDR). (C) Age-standardized disability-adjusted life-year (DALY) rate.

Supplementary Figure 3. Percentage of disability-adjusted life-years (DALYs) due to atrial fibrillation attributable to risk factors for 21 Global Burden of Disease regions, 2019, males.

Supplementary Figure 4. Percentage of disability-adjusted life-years (DALYs) due to atrial fibrillation attributable to risk factors for 21 Global Burden of Disease regions, 2019, females.

Supplementary Figure 5. Percentage of disability-adjusted life-years (DALYs) due to atrial fibrillation attributable to risk factors by age for both sexes, 2019

Supplementary Figure 6. Percentage of disability-adjusted life-years (DALYs) due to atrial fibrillation attributable to risk factors by age for males, 2019

Supplementary Figure 7. Percentage of disability-adjusted life-years (DALYs) due to atrial fibrillation attributable to risk factors by age for females, 2019
