# Supplementary figures and images for: Burden of atrial fibrillation and its attributable risk factors from 1990 to 2019: An analysis of the Global Burden of Disease study 2019
Source: Front Cardiovasc Med. 2022 Oct 26;9:997698. doi: 10.3389/fcvm.2022.997698 (PMC9643162; doi:10.3389/fcvm.2022.997698)

A

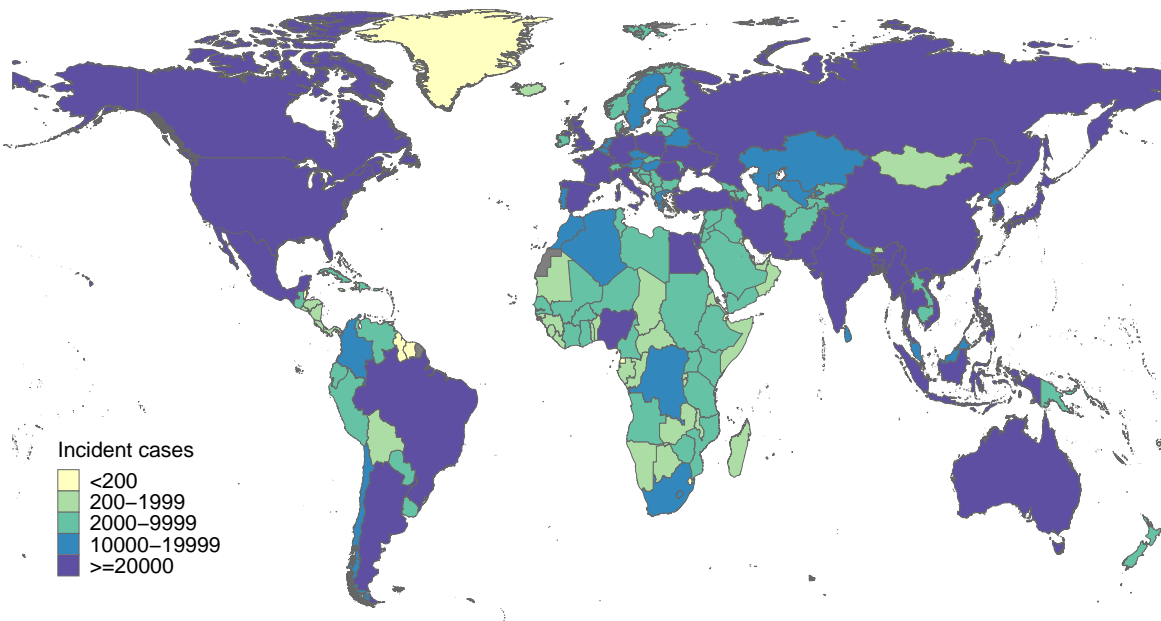

B

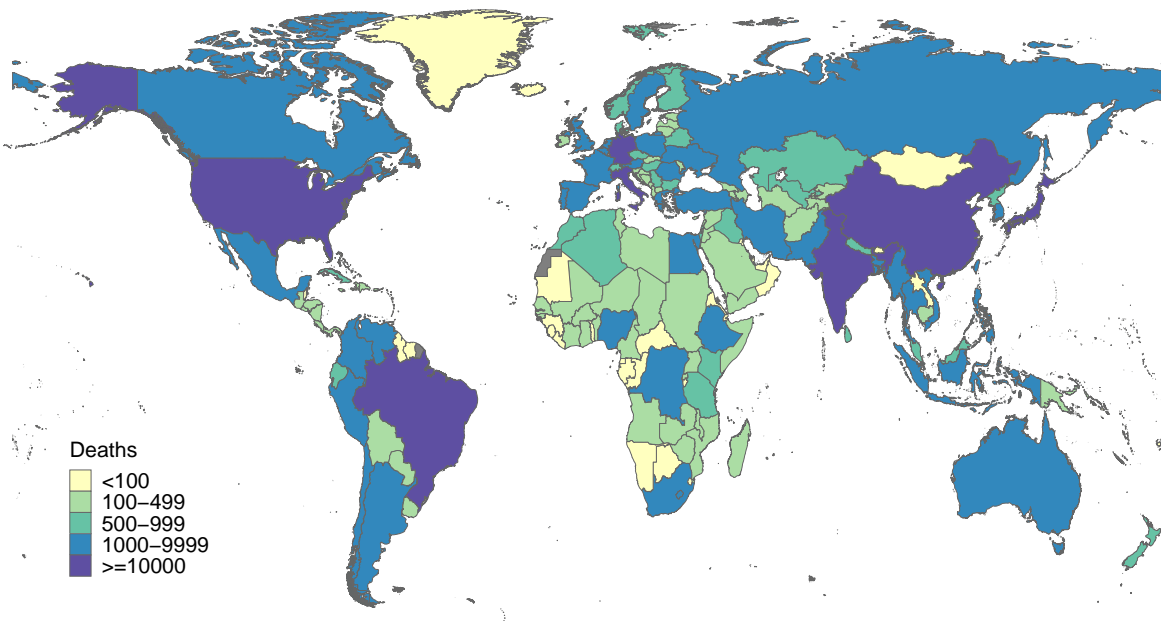

C

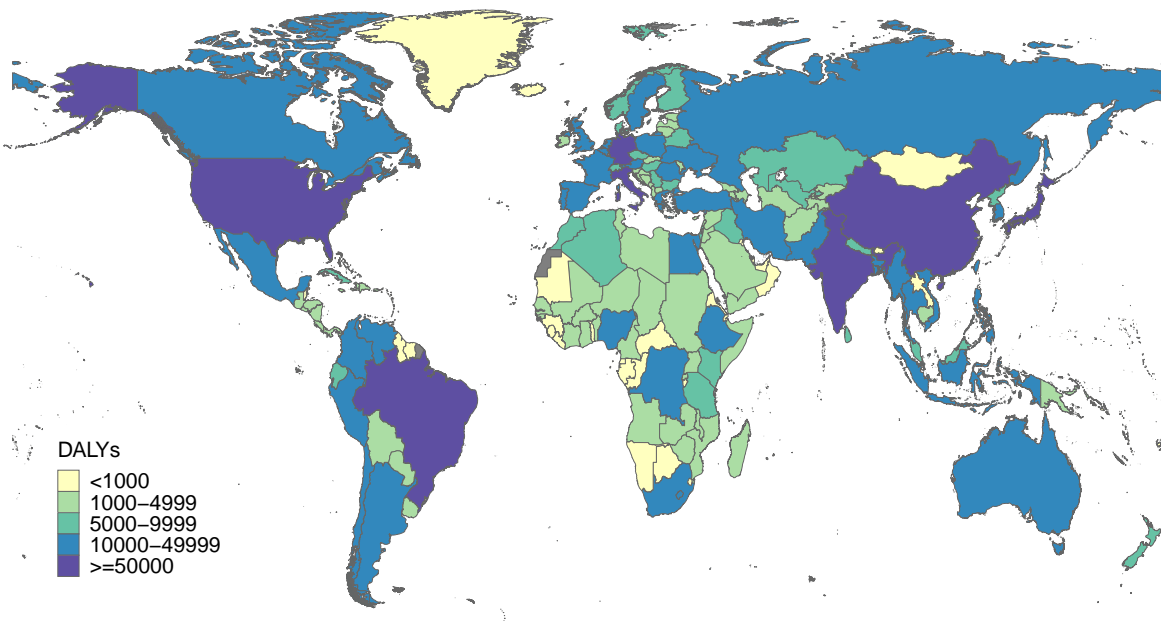

Supplement: Supplementary Figure 1 — The number of incident cases (A), death cases (B), and disability-adjusted life-years (DALYs) (C) of atrial fibrillation in 204 countries and territories in 2019. [file Image_1.PDF]

A

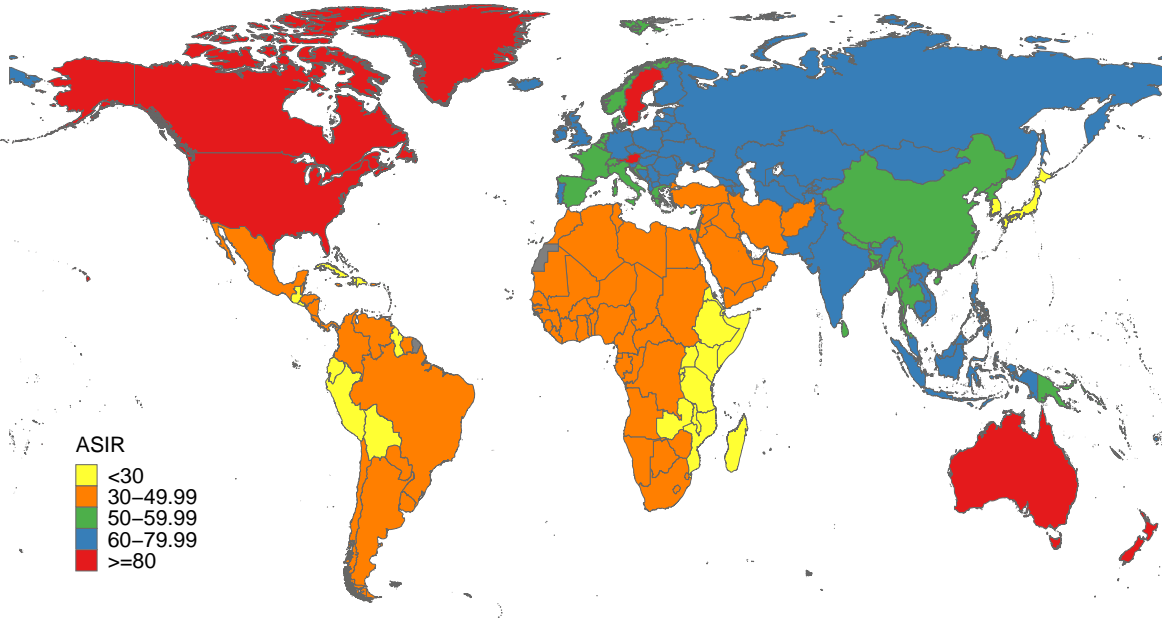

B

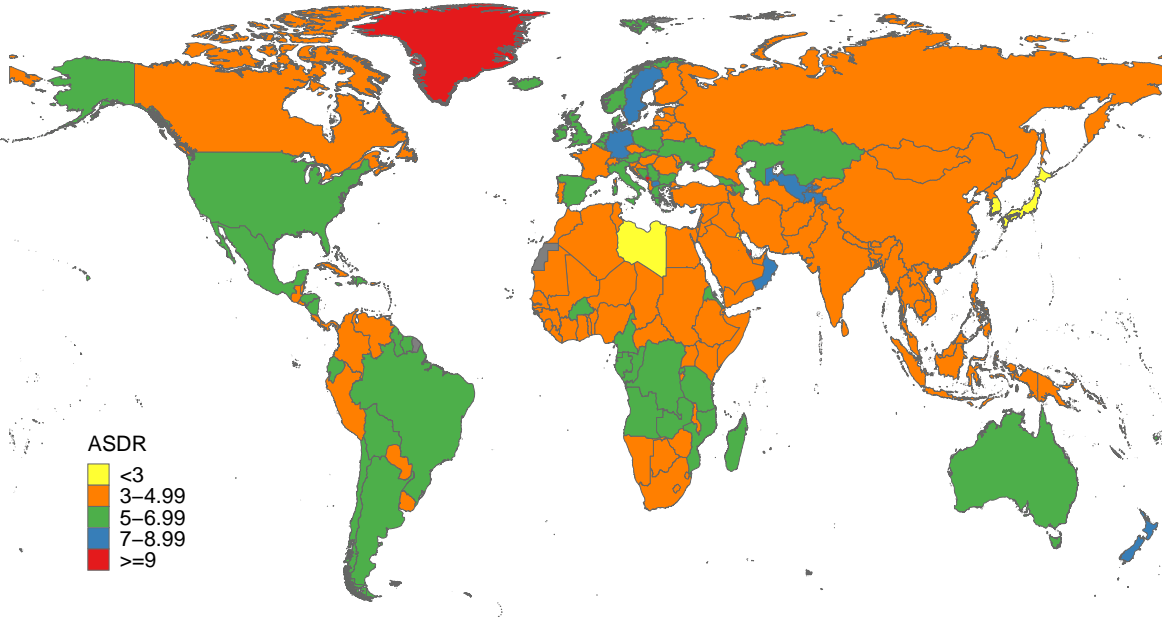

C

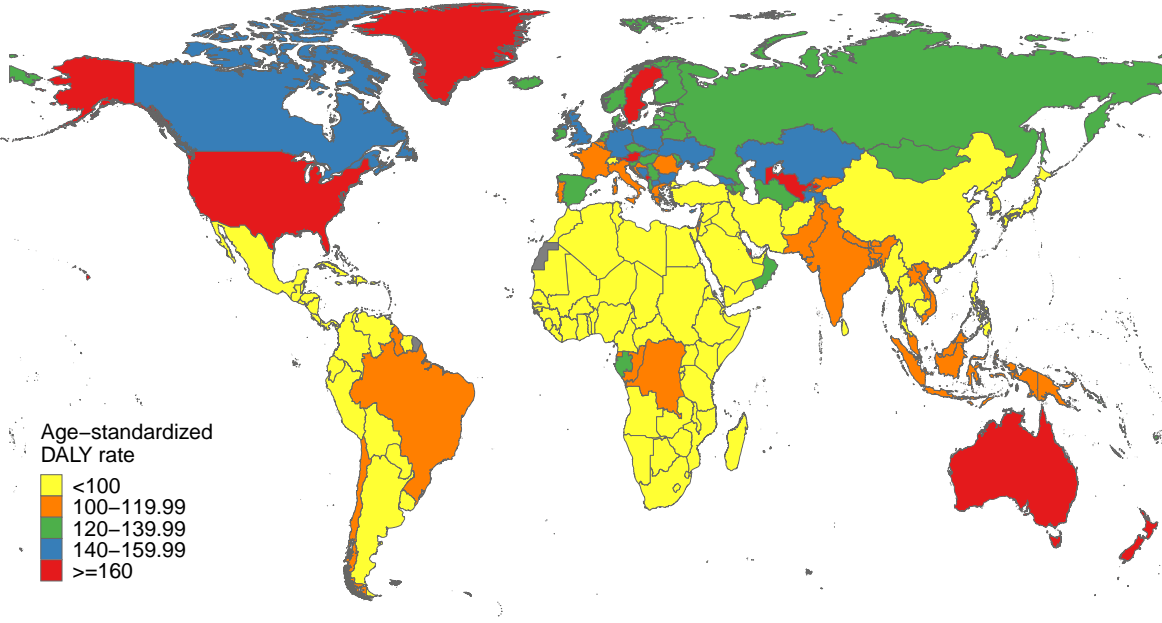

Supplement: Supplementary Figure 2 — The age-standardized rates of atrial fibrillation in 204 countries and territories in 2019. (A) Age-standardized incidence rate. (B) Age-standardized death rate (ASDR). (C) Age-standardized disability-adjusted life-year (DALY) rate. [file Image_2.PDF]

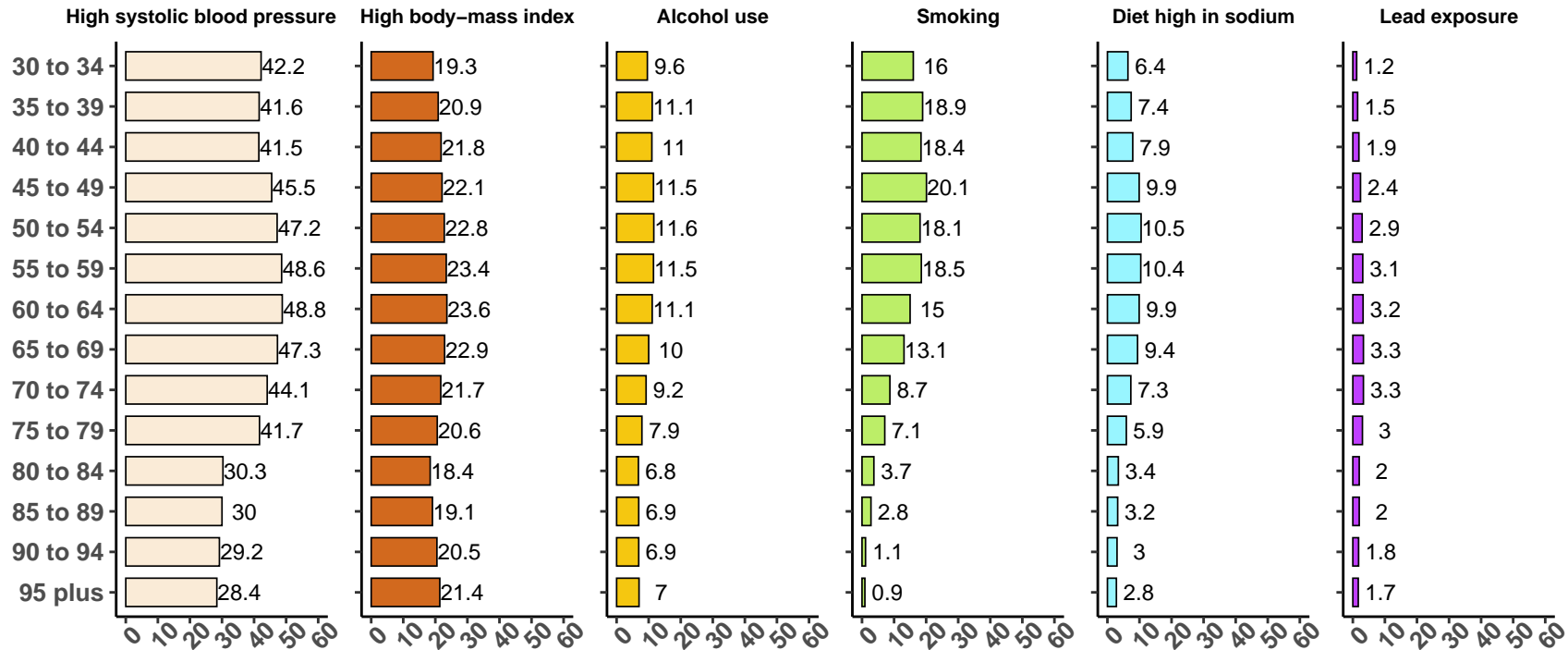

Supplement: Supplementary Figure 5 — Percentage of disability-adjusted life-years (DALYs) due to atrial fibrillation attributable to risk factors by age for both sexes, 2019. [file Image_5.PDF]

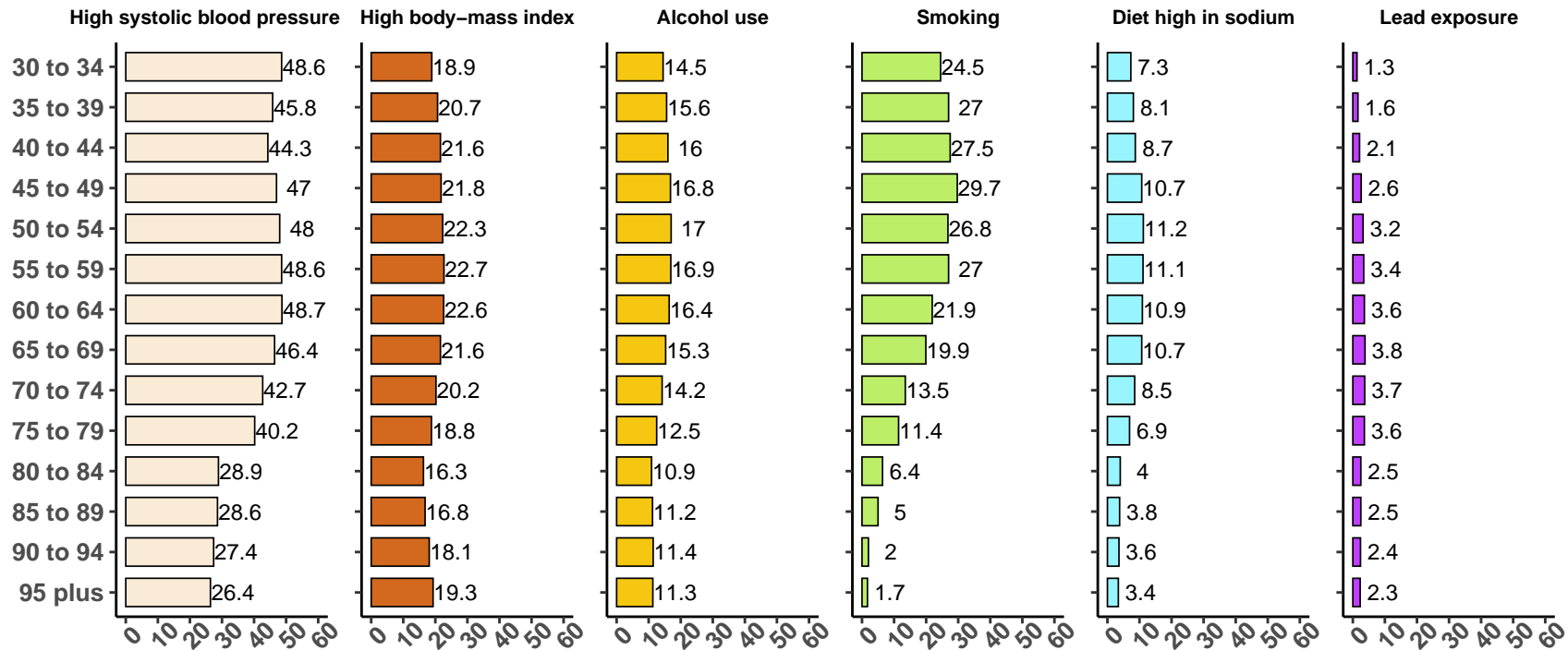

Supplement: Supplementary Figure 6 — Percentage of disability-adjusted life-years (DALYs) due to atrial fibrillation attributable to risk factors by age for males, 2019. [file Image_6.PDF]

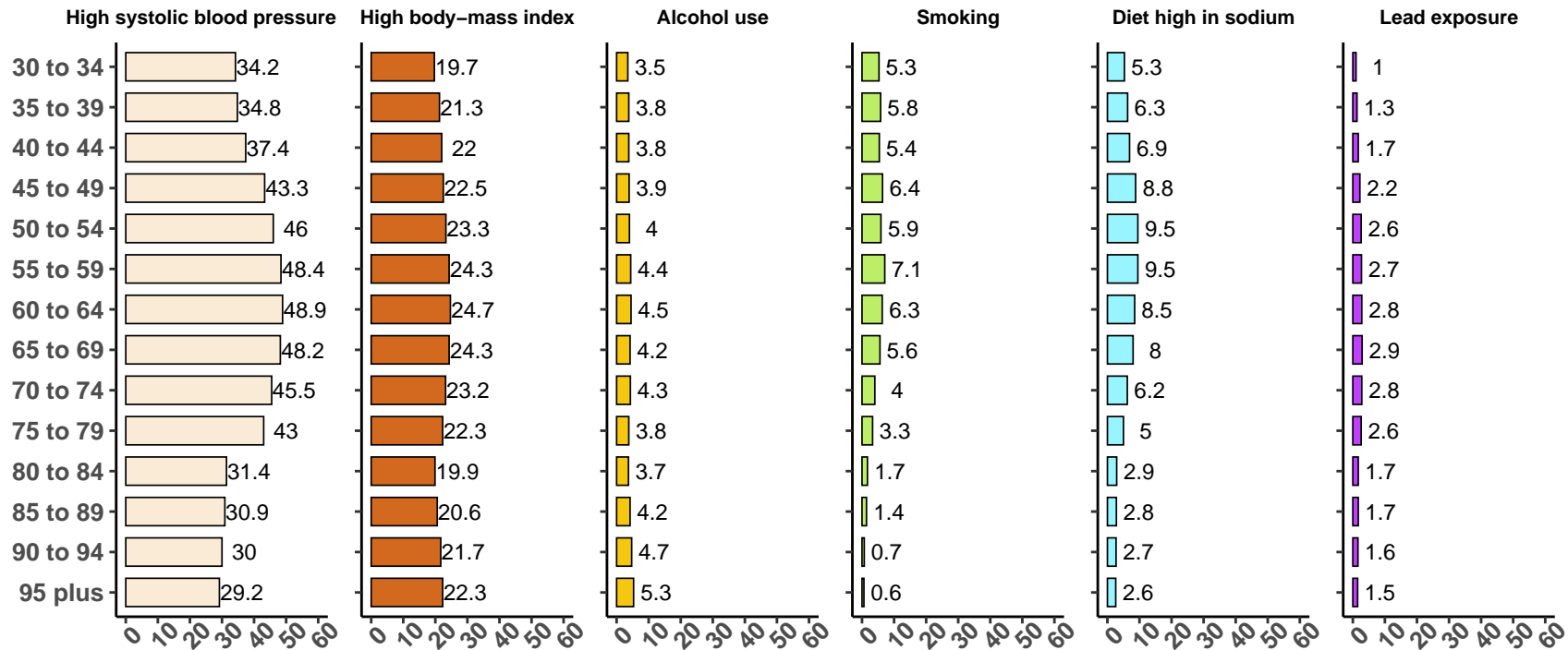

Supplement: Supplementary Figure 7 — Percentage of disability-adjusted life-years (DALYs) due to atrial fibrillation attributable to risk factors by age for females, 2019. [file Image_7.PDF]
