# Supplementary material for: Burden of atrial fibrillation and its attributable risk factors from 1990 to 2019: An analysis of the Global Burden of Disease study 2019
Source: Front Cardiovasc Med. 2022 Oct 26;9:997698. doi: 10.3389/fcvm.2022.997698 (PMC9643162; doi:10.3389/fcvm.2022.997698)
Supplement: Supplementary Figure 4 — Percentage of disability-adjusted life-years (DALYs) due to atrial fibrillation attributable to risk factors for 21 Global Burden of Disease regions, 2019, females. [file Image_4.PDF]

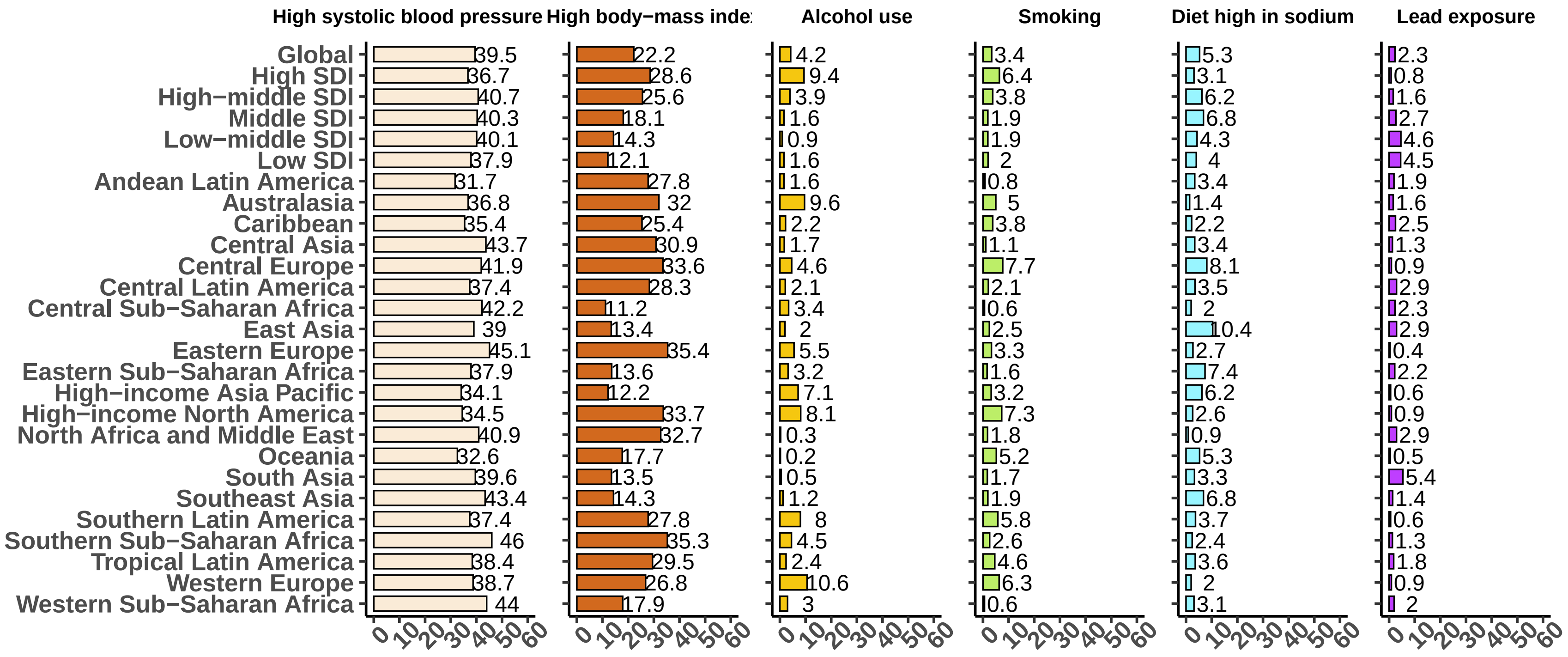

High systolic blood pressure
High body−mass inde
Alcohol use
Smoking
Diet high in sodium
Lead exposure
Global
High SDI
High−middle SDI
Middle SDI
Low−middle SDI
Low SDI
Andean Latin America
Australasia
Caribbean
Central Asia
Central Europe
Central Latin America
Central Sub−Saharan Africa
East Asia
Eastern Europe
Eastern Sub−Saharan Africa
High−income Asia Pacific
High−income North America
North Africa and Middle East
Oceania
South Asia
Southeast Asia
Southern Latin America
Southern Sub−Saharan Africa
Tropical Latin America
Western Europe
Western Sub−Saharan Africa
39.5 36.7 40.7 40.3 40.1 37.9 31.7 36.8 35.4 43.7 41.9 37.4 42.2 39 45.1 37.9 34.1 34.5 40.9 32.6 39.6 43.4 37.4 46 38.4 38.7 44
22.2 28.6 25.6 18.1 14.3 12.1 27.8 32 25.4 30.9 33.6 28.3 11.2 13.4 35.4 13.6 12.2 33.7 32.7 17.7 13.5 14.3 27.8 35.3 29.5 26.8 17.9
4.2 9.4 3.9 1.6 0.9 1.6 1.6 9.6 2.2 1.7 4.6 2.1 3.4 2 5.5 3.2 7.1 8.1 0.3 0.2 0.5 1.2 8 4.5 2.4 10.6 3
3.4 6.4 3.8 1.9 1.9 2 0.8 5 3.8 1.1 7.7 2.1 0.6 2.5 3.3 1.6 3.2 7.3 1.8 5.2 1.7 1.9 5.8 2.6 4.6 6.3 0.6
5.3 3.1 6.2 6.8 4.3 4 3.4 1.4 2.2 3.4 8.1 3.5 2 10.4 2.7 7.4 6.2 2.6 0.9 5.3 3.3 6.8 3.7 2.4 3.6 2 3.1
2.3 0.8 1.6 2.7 4.6 4.5 1.9 1.6 2.5 1.3 0.9 2.9 2.3 2.9 0.4 2.2 0.6 0.9 2.9 0.5 5.4 1.4 0.6 1.3 1.8 0.9 2
0 10 20 30 40 50 60
